# Divergent Cardiovascular Disease Burden in Hungary and Iraq: A Comparative Analysis of Global Burden of Disease Estimates

**DOI:** 10.3390/jcdd13100504

**Published:** 2026-10-08

**Authors:** Ahmed Lateef Abed Alkhaqani, Nóra Rozmann, Bence Schiszler, Miklós Novák, Bence Raposa

**Affiliations:** 1 Doctoral School of Health Sciences, Faculty of Health Sciences, University of Pécs, Vörösmarty Mihály Str. 4, 7621 Pécs, Hungary; miklos.novak@etk.pte.hu; 2 Institute of Emergency Care, Pedagogy of Health and Nursing Sciences, Faculty of Health Sciences, University of Pécs, Vörösmarty Mihály Str. 4, 7621 Pécs, Hungary; nora.rozmann@etk.pte.hu; 3 Institute of Basics of Health Sciences, Midwifery and Health Visiting, Faculty of Health Sciences, University of Pécs, Vörösmarty Mihály Str. 4, 7621 Pécs, Hungary; schiszler.bence@pte.hu

**Keywords:** cardiovascular disease, Global Burden of Disease, comparative epidemiology, incidence, mortality-to-incidence ratio, Hungary, Iraq

## Abstract

Background/objective: Cardiovascular disease (CVD) remains a major cause of death in Hungary and Iraq, which differ markedly in demographic structure, socioeconomic context, and health-system capacity. This study compared annual age-standardized cardiovascular mortality and incidence in Hungary and Iraq from 1990 to 2023 and examined selected risk-attributable cardiovascular mortality trajectories. Methods: The analyzed Global Burden of Disease (GBD) 2023 estimates for both sexes were used with annual age-standardized rates per 100,000 population. The primary temporal analysis used restricted cubic splines (RCS) for calendar year with four knots at the 5th, 35th, 65th and 95th percentiles, country effects, and country-by-spline interactions. Feasible generalized least squares with within-country AR (1) correlation were used to account for serial dependence. Selected-year predictions were obtained for 1990, 2000, 2010, 2020, and 2023. Risk-factor analyses were exploratory and limited to six prespecified country–risk pairs. Results: Age-standardized CVD mortality declined by 45.7% in Hungary and 21.1% in Iraq from 1990 to 2023, while age-standardized incidence declined by 43.9% and 15.4%, respectively. In 2023, CVD mortality was 289.2 per 100,000 in Hungary and 429.7 in Iraq, while incidence was 620.1 and 808.6 per 100,000, respectively. RCS analyses showed substantial non-linearity and country-specific trajectories for both mortality and incidence (all joint tests *p* < 0.001). In Hungary, attributable mortality rates declined for smoking, high LDL cholesterol, and air pollution; in Iraq, the BMI-attributable rate increased, while the fasting-plasma-glucose and high-systolic-blood-pressure trajectories showed more complex changes. Conclusions: Hungary and Iraq carry substantial but structurally different cardiovascular burdens. Iraq had the higher age-standardized CVD mortality and incidence in 2023, while Hungary experienced larger long-term declines. The trajectories indicate that cross-country differences reflect changing patterns of incident disease and risk-attributable burden rather than a single constant trend.

## 1. Introduction

Cardiovascular disease (CVD) remains the leading cause of death worldwide and continues to impose a major burden on populations and health systems. In the Global Burden of Disease (GBD) 2021 analysis, CVD accounted for 26.8% of all deaths globally, approximately one in four, with a global age-standardized mortality rate of 235 per 100,000 population and 612 million prevalent cases [1]. Although age-standardized mortality and disability-adjusted life-year (DALY) rates have fallen in many settings, the absolute burden has continued to rise because of population growth, population ageing, and persistent exposure to modifiable risk factors. Projections built on GBD 2019 estimate that annual cardiovascular deaths will rise from 20.5 million in 2025 to 35.6 million in 2050, even as age-standardized mortality falls by approximately 30% [2]. Ischaemic heart disease and stroke remain the dominant contributors, and their impact is shaped by the interaction between metabolic risk, behavioural exposure and unequal access to prevention and treatment [3].

Low- and middle-income settings bear a disproportionate share of global cardiovascular mortality and frequently have more limited access to effective prevention and treatment, even though age-standardized rates have improved in many regions [4,5]. In North Africa and the Middle East, CVD remains a major cause of mortality and morbidity, with high systolic blood pressure, dietary risks, and high LDL cholesterol representing the leading attributable risk factors [6]. Evidence from the Prospective Urban Rural Epidemiology (PURE) study indicates that approximately 70% of cardiovascular disease cases and deaths are attributable to 14 modifiable risk factors, although their relative importance varies across country-income groups [7].

National cardiovascular profiles therefore cannot be explained by biology alone, but must be interpreted in relation to social conditions, epidemiological transition and health-system performance. However, epidemiological patterns, underlying drivers, healthcare system capacity, and policy responses to CVD vary significantly across regions and countries [6]. Hungary and Iraq exemplify two profoundly distinct instructive cases of cardiovascular disease burden: Hungary is a critical outlier within the European Union and OECD contexts despite its developed-nation status and advanced healthcare infrastructure, whereas Iraq represents a conflict-affected nation within the Middle East and North Africa (MENA) region, facing compounded cardiovascular challenges stemming from decades of healthcare system disruption, economic constraints, and rapid epidemiological transition [6,8].

Hungary and Iraq are instructive because they differ on nearly every dimension that shapes those profiles. Hungary combines universal health coverage and established medical infrastructure with persistently high cardiovascular mortality and marked internal inequality; circulatory diseases account for a large share of Hungarian mortality despite gradual and long-term improvements [7,8]. Hungarian evidence indicates that cardiovascular risk is concentrated in socially disadvantaged populations. In a screening program covering 6211 residents of underprivileged settlements, 72% had a body mass index above 25 kg/m^2^, more than 90% exercised for fewer than three hours per week, and active smoking reached 60.3% among self-identified Roma participants compared to 28.7% among non-Roma participants in the same settlements [9]. The estimated ten-year cardiovascular risk is correspondingly higher in the Hungarian Roma population than in the general population [10]. Therefore, Hungary is analytically important not only as a high-burden European setting but also as an example of how an elevated cardiovascular burden can persist within a developed health system when risk reduction is distributed unevenly.

Iraq provides a contrasting context. Non-communicable diseases have become a dominant component of the Iraqi disease burden, and CVD remains the leading cause of death in both sexes. Between 2003 and 2021, the age-standardized incidence of diabetes and kidney disease rose by 42.9%, while overall non-communicable disease prevalence remained broadly flat [11]. Hospital-based Iraqi evidence describes a young clinical presentation of acute coronary syndrome, with a mean age of 56.2 years, eight to ten years younger than Western counterparts, with high frequencies of smoking, hyperlipidemia, hypertension, and diabetes among admitted patients [12]. This pattern is shaped not only by conventional cardiometabolic risk but also by prolonged health-system disruption, incomplete surveillance, and the wider consequences of conflict and instability: countries in the region with a history of war or ongoing conflict show consistently higher cardiovascular death, DALY, and incidence rates than more stable neighbors [13].

Comparing these two countries within a single analytic framework offers an opportunity to describe how cardiovascular incidence, mortality, and risk-attributable burden have evolved under markedly different demographic and health-system contexts. Therefore, this study aimed to (i) quantify and compare annual age-standardized cardiovascular mortality and incidence in Hungary and Iraq between 1990 and 2023 for overall CVD and selected component causes; (ii) characterize non-linear temporal trajectories using restricted cubic splines; (iii) compare the fitted trajectories of selected risk-attributable cardiovascular mortality rates with the corresponding overall CVD mortality trajectories; and (iv) examine mortality-to-incidence ratios as ecological descriptive proxies while avoiding individual-level causal interpretation.

## 2. Materials and Methods

### 2.1. Study Design

This study is a comparative secondary analysis of GBD 2023 estimates for Hungary and Iraq, accompanied by a structured contextual synthesis of the recent literature. The quantitative component provides standardized, internally consistent country-level estimates across time and disease categories; the literature synthesis situates these estimates in relation to risk factors, health systems, and epidemiological transition, which the modelled estimates alone cannot do.

This design was selected because the research question required both a standardized quantitative comparison across countries and a contextual interpretation of recent evidence on risk factors, health systems, and epidemiological transitions. Comparable population-level estimates are of limited value for policy unless they are read against the settings that generate them [14].

### 2.2. Data Source and Extraction

The GBD study produces comprehensive and internally comparable metrics of disease burden over time, covering incidence, prevalence, mortality, years of life lost (YLLs), years lived with disability (YLDs) and DALYs. All estimates analyzed here are drawn from GBD 2023, produced by the Institute for Health Metrics and Evaluation (IHME); the underlying input data are catalogued at https://ghdx.healthdata.org/gbd-2023 (accessed on 30 May 2026).

Estimates were obtained from the Global Burden Disease Study 2023 through the Global Health Data Exchange and GBD Results Tool [15]. The GBD 2023 provides estimates for 292 causes of death and 375 diseases and injuries, together with the risk-attributable burden for 88 risk factors, for 204 countries and territories for each year from 1990 to 2023 [16]. Mortality was estimated using the Cause of Death Ensemble model applied to vital registration, verbal autopsy, survey, census, surveillance, and registry data. Non-fatal outcomes were estimated using DisMod-MR 2.1, and the risk-attributable burden was estimated within the comparative risk assessment framework.

The input data for GBD 2023 comprise vital registration coded to the International Classification of Diseases, supplemented by household mortality surveys where registration is incomplete; cohort studies, registries, population surveys, and administrative data for incidence and prevalence; and population-representative surveys, surveillance systems, and geospatial Gaussian process regression models for risk factor exposure. DALYs are computed as the sum of YLLs, referenced to a maximum observed life expectancy, and YLDs, weighted by standardized disability weights for each health state.

Within the GBD cause hierarchy, CVD is a Level 2 cause within the Level 1 non-communicable disease group, and is subdivided at Level 3 into rheumatic heart disease, ischemic heart disease, stroke, hypertensive heart disease, non-rheumatic valvular heart disease, cardiomyopathy and myocarditis, atrial fibrillation and flutter, aortic aneurysm, lower-extremity peripheral arterial disease, endocarditis, pulmonary arterial hypertension, and other cardiovascular and circulatory diseases. The annual disease extraction used both sexes, age-standardized rates, and the GBD rate metric per 100,000 population for deaths and incidence. The complete annual Level 3 extraction represented in the supplied exports comprised 13 cause series for mortality and 10 cause series for incidence; incidence is not available for aortic aneurysm, hypertensive heart disease, or other cardiovascular and circulatory diseases.

All analyses were restricted to 1990–2023, covering the full span of the GBD 2023 cause-specific estimates. Data were extracted for Hungary and Iraq, together with global and regional estimates used solely for contextual benchmarking. Within the GBD location hierarchy, Iraq is classified in the North Africa and Middle East region, whereas Hungary is classified in Central Europe. The principal country comparison used annual age-standardized rates per 100,000 population for both sexes from 1990 to 2023. Absolute numbers and crude all-age rates were used only for describing the global population burden and in the supplementary population-scale analyses. Incidence estimates were unavailable for aortic aneurysm, hypertensive heart disease, and other cardiovascular and circulatory diseases. Consequently, the mortality-to-incidence ratio analysis included the aggregate CVD category and nine component causes for which both mortality and incidence estimates were available. For the risk factor component, GBD 2023 estimates of the change in cardiovascular burden attributable to major modifiable risks were extracted for both countries for the period 1990–2023, covering 14 risk exposure items: smoking, tobacco (the Level 2 parent category), high systolic blood pressure, high fasting plasma glucose, high body mass index, high LDL cholesterol, kidney dysfunction, low physical activity, dietary risks, diet high in sodium, air pollution, non-optimal temperature, high alcohol use, and other environmental risks. Three metrics were obtained for each: change in the absolute number of attributable deaths or DALYs, change in the attributable rate per 100,000, and change in the attributable fraction, each with 95% uncertainty intervals. Smoking is reported in the main comparison and tobacco in the table footnote to avoid presenting a risk and its parent category as independent rows.

### 2.3. Age Standardization and Age-Structure Considerations

Age-standardized rates are the principal country comparison metric because Hungary and Iraq have markedly different age distributions. Age-standardized estimates were extracted directly from the GBD 2023 Results Tool for both sexes for each year from 1990 to 2023. The previous crude, all-age analyses were retained only as supplementary descriptive material because crude rates are strongly influenced by the population age structure and do not provide a clean comparison of the underlying age-independent cardiovascular risk.

Crude rates remain relevant for describing the burden of health systems because they represent the observed number of events relative to the total population. However, cardiovascular event rates increase steeply with age, and Hungary and Iraq differ profoundly in terms of population age structure [17]. Accordingly, crude results are presented as a supplementary description of the population burden rather than as the primary measure of cross-country cardiovascular risk [18]. The standard population underlying the GBD age-standardized estimates is not numerically interchangeable with the European Standard Population used by Eurostat [19]; therefore, Eurostat comparisons were used only for contextual interpretation. When external standardized benchmarks were quoted, the relevant standard population was stated. No external age-standardized values were substituted for the directly extracted GBD 2023 Hungary or Iraq estimates.

### 2.4. Contextual Literature Synthesis

Peer-reviewed and institutional sources published primarily between 2020 and 2026 were sought, with priority given to systematic reviews, large observational studies, pooled multi-country analyses, and major institutional reports addressing cardiovascular burden, risk factors, or the health-system context in Hungary, Iraq, or the wider Middle East and North Africa region. Earlier authoritative national or institutional sources were retained where they provided uniquely relevant information not available from more recent sources. Searches were run in PubMed, Scopus, Web of Science and Google Scholar, combining terms for cardiovascular disease, mortality, incidence, prevalence, risk factors, Hungary, Iraq, epidemiology, health systems and avoidable mortality. Targeted searches were run for institutional sources from the World Health Organization, the OECD, Eurostat and the Institute for Health Metrics and Evaluation.

Sources were eligible if they reported cardiovascular mortality, incidence, or prevalence, major cardiovascular risk factors, health-system context, or avoidable mortality in Hungary or Iraq. Where Iraq-specific evidence was sparse, regional evidence from North Africa and the Middle East was used for context and labelled as such. Hospital-based clinical series are used only to describe clinical presentation and are never used to estimate population prevalence or exposure.

### 2.5. Statistical Analysis

Descriptive summaries are reported for the global burden, cause-specific mortality, cause-specific incidence, and risk-attributable burden. Because GBD estimates are model-based and serially dependent rather than independent observations, no significance tests are applied to country-year series, and no *p*-values are reported for trend comparisons. Uncertainty is reported as UIs reflecting estimation uncertainty.

The primary temporal analysis used the complete annual age-standardized series from 1990 to 2023. For the four prespecified outcomes, models were fitted on the natural-log scale of the rate to preserve positivity. The calendar year was represented by a restricted cubic spline with four knots placed at the 5th, 35th, 65th, and 95th percentiles of the observed year distribution (1991.65, 2001.55, 2011.45, and 2021.35. The combined model included country, spline terms for calendar year, and country-by-spline interaction.

Serial dependence across adjacent annual GBD estimates was accounted for using feasible generalized least squares with an AR (1) covariance structure within country. For each principal outcome, we report the overall likelihood-ratio test for calendar-year terms, the joint test for non-linearity, and the joint country-by-spline interaction test. Non-linearity was assessed by comparing the full spline model with a model retaining country-specific linear temporal trends; the interaction test compared the full interaction model with an otherwise identical model without country-by-spline interaction. Because annual GBD estimates are model-based posterior estimates rather than independent observations, these regression tests and confidence bands are presented as model-based descriptive summaries.

Predicted rates and model-based 95% confidence intervals were obtained from the fitted covariance matrix and back-transformed to the rate scale for 1990, 2000, 2010, 2020, and 2023. Annual observed GBD 95% uncertainty intervals were retained and are shown on the figures for visual reference. Local maxima and minima identified from the derivative of the fitted spline are reported as descriptive features of the fitted curves rather than as formal epidemiological breakpoints.

The six selected risk-factor series were analyzed separately within the country using the same four-knot RCS specification and country-specific AR (1) covariance. The fitted trajectories were descriptively compared with the corresponding overall CVD mortality trajectories. To facilitate visual comparison despite different absolute magnitudes, fitted risk trajectories were indexed to their respective 1990 fitted values (1990 = 100). No causal mediation analysis was performed, and no risk-attributable estimates were obtained.

The mortality-to-incidence ratio (MIR) was retained as a secondary ecological proxy and recalculated using age-standardized annual rates. For each cause and country, the MIR was the mean mortality rate divided by the mean incidence rate over 1990–2023. A higher MIR indicates more deaths relative to the estimated incidence in the observation period, but it is not a probability of death after an incident case and is particularly difficult to interpret when incidence and mortality are separated by long time intervals. The former cross-cause correlation analysis was retained only in the supplementary dataset as a sensitivity description. For transparency, the original five-year period-mean analysis and original crude MIR/correlation outputs are retained in the Supplementary Material. They are not used for the principal temporal conclusions.

### 2.6. Ethical Considerations

This study used publicly available aggregated secondary estimates and the published literature. Ethical approval was not required, and no identifiable individual-level data were used in this study. All sources have been cited and acknowledged.

### 2.7. Data Availability

All estimates are publicly available from the GBD Results Tool (https://vizhub.healthdata.org/gbd-results/ (accessed on 30 May 2026)). The supplementary package contains the annual analysis dataset, model outputs, selected year predictions, figures, and complete statistical syntax used for the revised analyses.

## 3. Results

### 3.1. Global Context of Cardiovascular Disease

**Figure 1:**
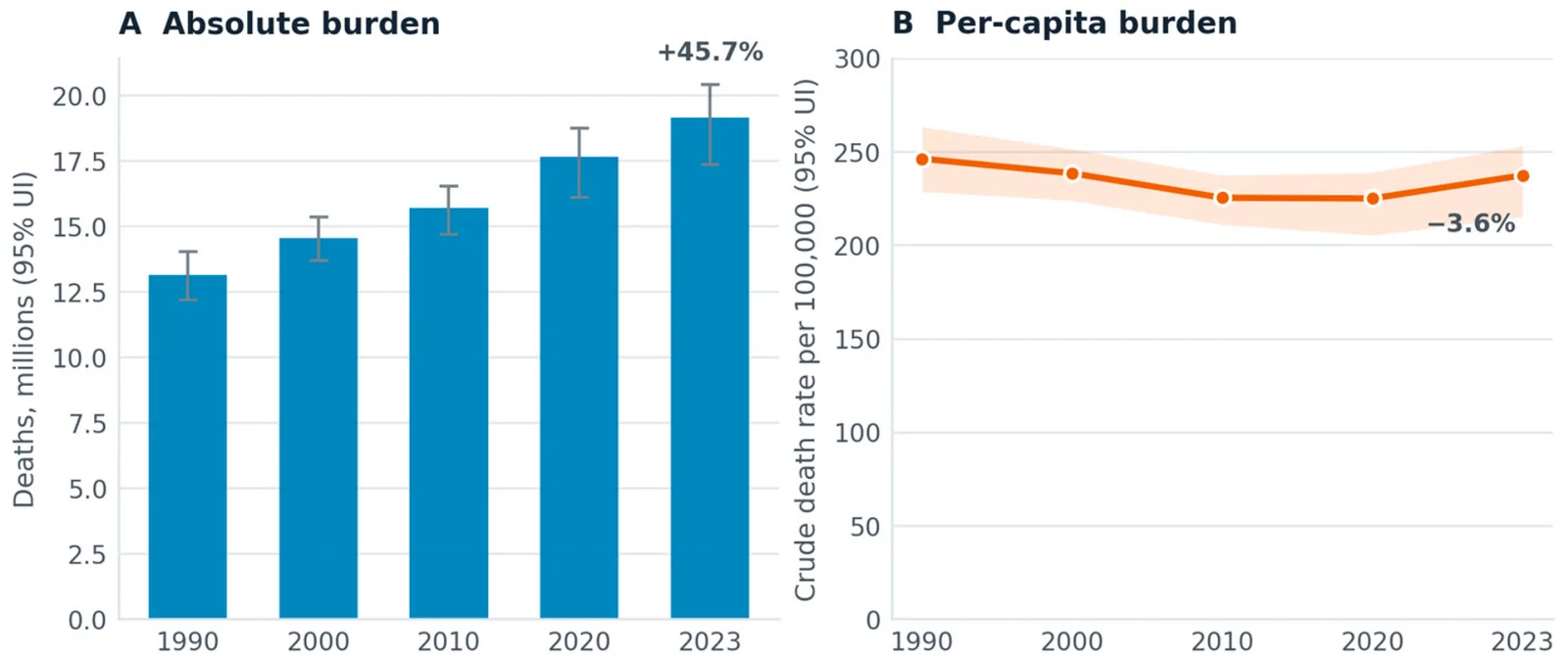
Global Cardiovascular Disease Burden, 1990–2023. (**A**) Absolute deaths in millions, with 95% uncertainty intervals. (**B**) Crude death rate per 100,000 population with a 95% uncertainty interval. Source: Authors’ analysis of the GBD 2023 estimates obtained from the GBD Results Tool.

Global cardiovascular deaths increased from 13.14 million (95% UI 12.20–14.04) in 1990 to 19.14 million (17.36–20.40) in 2023, an increase of 6.00 million deaths or 45.7% (Table 1, Figure 1A). Over the same period, the crude global death rate per 100,000 fell slightly from 246.2 to 237.3 (−3.6%; Figure 1B). Because the world population aged substantially across these three decades, a falling crude rate implies a larger fall in the age-standardized rate; therefore, the growth in absolute burden is driven by population growth and aging rather than by a uniform worsening of per-capita risk. This is consistent with the GBD 2023, which reported rising absolute DALY counts alongside a 12.6% fall in age-standardized DALY rates between 2010 and 2023.

### 3.2. Annual Age-Standardized Cardiovascular Burden

**Figure 2:**
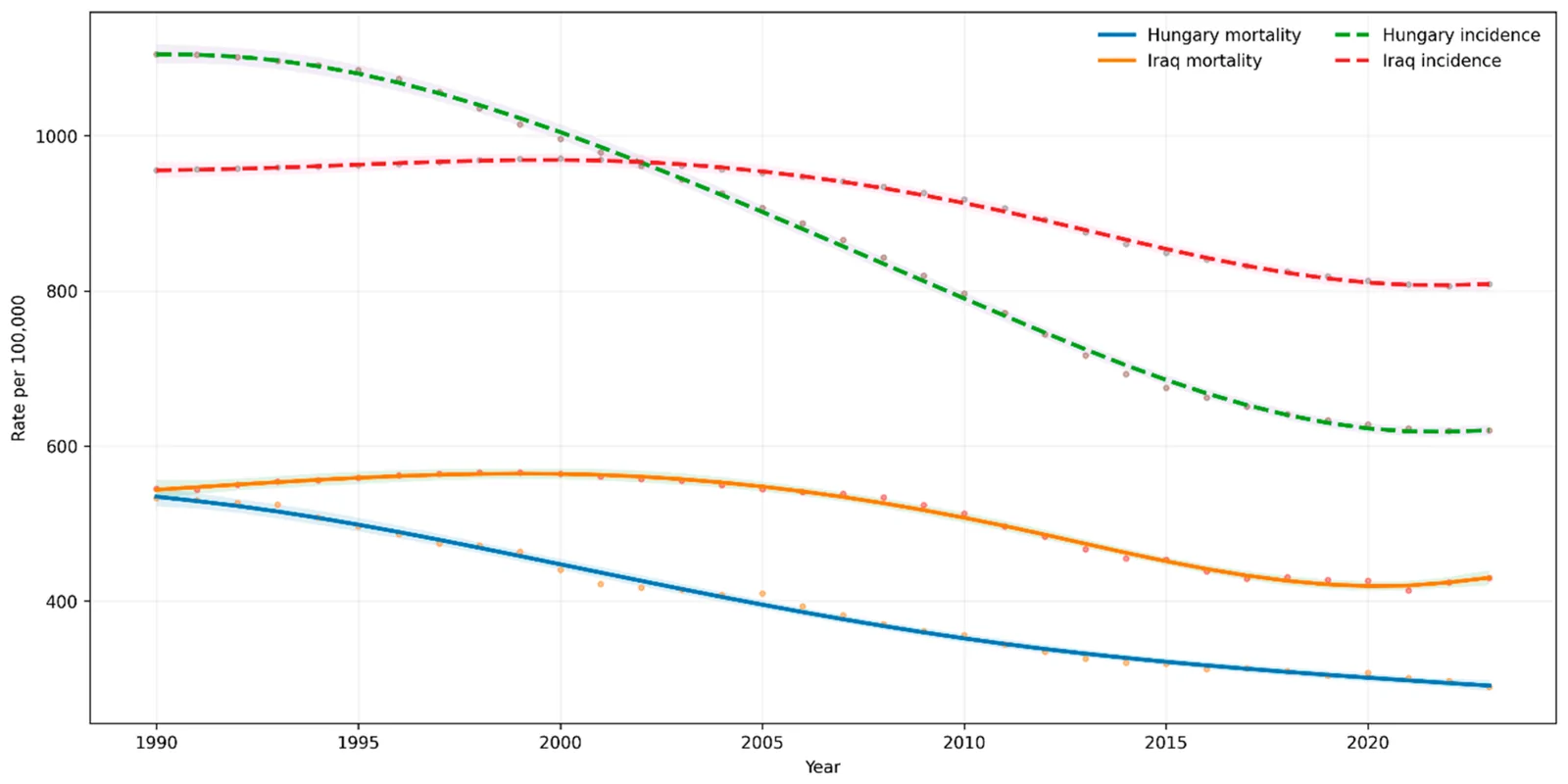
Age-Standardized Cardiovascular Mortality and Incidence in Hungary and Iraq, 1990–2023 with Restricted Cubic Spline Estimates. The solid and dashed lines represent mortality and incidence, respectively. Shading represents model-based 95% confidence intervals around the fitted RCS trajectories; markers/whiskers show annual GBD 2023 estimates with 95% uncertainty intervals in selected years for readability. Source: Authors’ analysis of the GBD 2023 estimates obtained from the GBD Results Tool.

The principal comparison used the annual age-standardized GBD 2023 estimates for each year from 1990 to 2023. The observed CVD mortality rate declined from 533.0 to 289.2 per 100,000 individuals in Hungary (−45.7%) and from 544.8 to 429.7 per 100,000 individuals in Iraq (−21.1%). The age-standardized CVD incidence declined from 1105.3 to 620.1 per 100,000 in Hungary (−43.9%) and from 955.5 to 808.6 per 100,000 in Iraq (−15.4%). Thus, the apparent cross-country ordering seen in crude all-age rates changed materially after standardization: by 2023, Iraq had higher age-standardized mortality and incidence rates. The fitted annual trajectories are shown in Figure 2. Table 2 presents the model-based spline predictions for the selected years requested by the reviewer. These values were estimated from the fitted RCS models and were distinct from the annual GBD uncertainty intervals.

### 3.3. Restricted Cubic Spline Analysis of Principal Outcomes

All eight principal outcome series (four causes × two measures) showed evidence of a calendar-year association and non-linear temporal structure, and the country-specific trajectories differed materially. The overall calendar-year likelihood-ratio statistics, joint non-linearity tests, and country-by-spline interaction tests are presented in Table 3. For all-CVD mortality, the calendar-year LR statistic was 293.51 (10 degrees of freedom, *p* < 0.001), the non-linearity LR statistic was 122.87 (8 degrees of freedom, *p* < 0.001), and the country-by-spline interaction LR statistic was 164.53 (5 degrees of freedom, *p* < 0.001). For all-CVD incidence, the overall calendar-year likelihood-ratio statistic was χ^2^ = 339.43 (10 df, *p* < 0.001), the joint non-linearity statistic was χ^2^ = 174.56 (8 df, *p* < 0.001), and the country-by-spline interaction statistic was χ^2^ = 244.88 (5 df, *p* < 0.001). Table 3 presents the complete model-based tests for the four prespecified cardiovascular outcomes. The common knots were 1991.65, 2001.55, 2011.45 and 2021.35. The AR (1) coefficients were country- and outcome-specific and are reported in the supplementary model output table.

### 3.4. Cause-Specific Age-Standardized Trajectories

**Figure 3:**
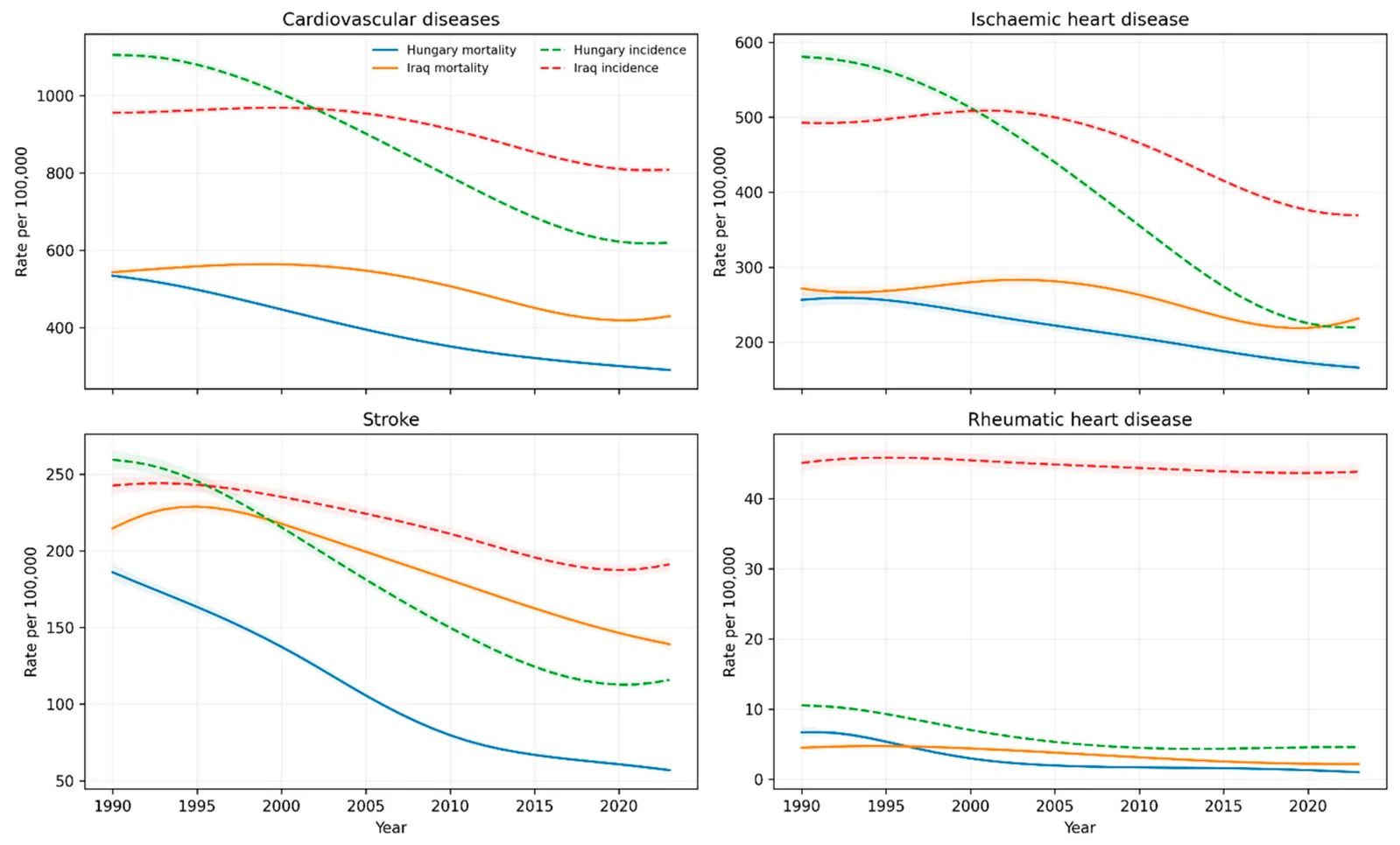
Restricted Cubic Spline Trajectories for Annual Age-Standardized Mortality and Incidence of All CVD, Ischemic Heart Disease, Stroke, and Rheumatic Heart Disease in Hungary and Iraq, 1990–2023. The shaded area represents the model-based 95% confidence intervals. Source: Authors’ analysis of the GBD 2023 estimates obtained from the GBD Results Tool.

The cause-specific RCS results showed the same broad pattern of non-linearity, but with different magnitudes and timing. Age-standardised mortality declined from 1990 to 2023 for ischemic heart disease (−35.4% in Hungary; −14.8% in Iraq), stroke (−69.6%; −35.5%), and rheumatic heart disease (−84.8%; −51.5%). Incidence also declined for all three prespecified causes in Hungary and for ischemic heart disease and stroke in Iraq, whereas the incidence of rheumatic heart disease in Iraq changed only slightly over the period (−2.8%). The complete endpoint values are shown in Table 4, and the fitted cause-specific trajectories are displayed in Figure 3.

These endpoint comparisons describe the net change but do not assume constant trends. For example, the RCS identified an early mortality maximum for Iraq around 1999 for all CVD and late-period minima around 2020–2022 for several outcomes; cause-specific local extrema are reported in the supplementary turning point table.

### 3.5. Exploratory Risk-Factor Trajectories

The risk factor extension was intentionally limited to six prespecified country–risk pairs. In Hungary, the observed age-standardized CVD mortality rate attributable to smoking declined by 65.5% from 80.23 to 27.67 per 100,000; high LDL cholesterol by 58.3% from 142.13 to 59.21; and air pollution by 76.3% from 119.52 to 28.33. In Iraq, the BMI-attributable rate increased by 30.0% from 60.63 to 78.84 per 100,000, and the high fasting plasma glucose increased by 9.9% from 61.69 to 67.77, whereas the high systolic blood pressure-attributable rate decreased by 18.4% from 328.96 to 268.36. All six fitted trajectories showed evidence of non-linearity (joint *p* < 0.001), but the timing and shape of the change differed across the risks and countries. The corresponding 1990 and 2023 values and observed changes are summarized in Table 5.

**Figure 4:**
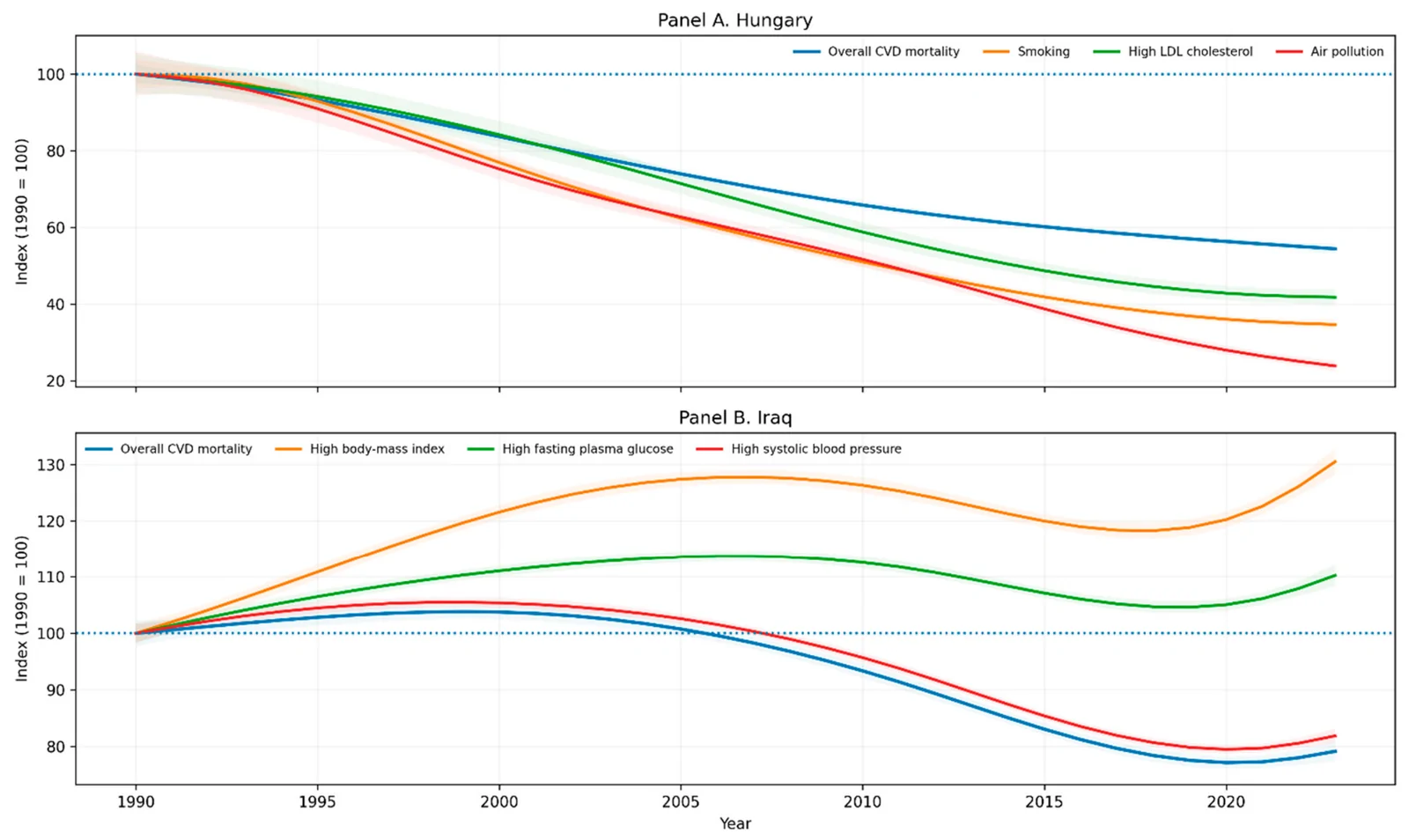
Indexed trajectories (1990 = 100) of overall CVD mortality and selected risk-attributable CVD mortality rates. Panel (**A**): Hungary—overall CVD mortality, smoking, high LDL cholesterol and air pollution. Panel (**B**): Iraq, overall CVD mortality, high body-mass index, high fasting plasma glucose and high systolic blood pressure. The figure is descriptive and does not represent a causal mediation analysis. Source: Authors’ analysis of GBD 2023 estimates obtained from the GBD Results Tool.

The risk trajectories are shown as indices relative to 1990 in Figure 4, allowing a comparison of the direction and timing rather than the absolute magnitude. In Iraq, the fitted BMI-attributable trajectory peaked around 2006.7 before reaching a local minimum around 2017.6 and rising again, toward 2023. The glucose trajectory showed a similar broad turning-point pattern. In Hungary, the smoking and LDL-attributable burdens declined across most of the study period, with late-period flattening. These are descriptive co-trajectories and do not demonstrate causal relationships.

### 3.6. Mortality-to-Incidence Ratio as a Secondary Ecological Proxy

Using age-standardised annual rates, the mean CVD mortality-to-incidence ratio over 1990–2023 was approximately 0.459 in Hungary and 0.559 in Iraq. For ischemic heart disease, the corresponding ratios were approximately 0.533 and 0.560, and for stroke, 0.608 and 0.873. These ratios are ecological summaries rather than individual-level case-fatality probabilities; mortality may reflect cases accumulated in earlier periods, and incidence and mortality are not linked at the individual level. The complete cause-specific MIR table is provided in the Supplementary Material, together with the original crude MIR as a sensitivity description.

### 3.7. Supplementary Crude Analysis and Population-Scale Burden

The previously reported crude all-age analyses are retained in the Supplementary Material rather than used for the principal temporal conclusions. They remain useful for describing population-scale health system burden and for showing how population growth can produce rising absolute numbers despite stable or declining rates. Global estimates are likewise retained only as descriptive context and are not included in the Hungary–Iraq comparative RCS analyses.

## 4. Discussion

### 4.1. Principal Findings

The revised age-standardized analysis changes the interpretation of the Hungary–Iraq comparison. In 1990, the age-standardized CVD mortality rates were similar in Hungary and Iraq (533.0 and 544.8 per 100,000, respectively), but by 2023, Hungary had a substantially lower rate (289.2 versus 429.7). Incidence showed the same reversal in relative position: Hungary was higher in 1990 (1105.3 versus 955.5) but lower in 2023 (620.1 versus 808.6). Hungary therefore experienced larger long-term reductions in both mortality and incidence. The RCS models further demonstrate that these changes cannot be adequately represented by a single constant annual trend: calendar-year association was strong, non-linearity was present, and country-by-spline interactions were substantial for both mortality and incidence. These findings suggest that both countries carry a substantial cardiovascular burden, but the structure of this burden differs in important ways. A key finding of the analysis is that Hungary showed consistently higher mortality and incidence for most cardiovascular causes, whereas Iraq showed lower overall rates but a less favorable pattern for several metabolic and environmental risk drivers.

The highest global cardiovascular burden was observed in 2023, when estimated deaths reached 19.14 million, whereas the crude death rate per 100,000 population remained below the 1990 value. This finding suggests that the growth in the global cardiovascular burden has been driven largely by population growth and aging rather than by a uniform worsening of per-capita risk. This interpretation is consistent with recent global analyses showing that absolute cardiovascular deaths continue to rise despite declines in age-standardized mortality and disability-adjusted life year rates [1,2]. In contrast, some regional analyses emphasize improvements in age-standardized rates and may therefore appear inconsistent with the present findings [6]. The use of different epidemiological metrics best explains this difference, because crude counts reflect demographic expansion, whereas age-standardized rates are intended to isolate the underlying risk independent of population structure.

The observed divergence in incidence likely reflects several interacting, population-level pathways. In Hungary, long-term reductions in smoking- and air pollution-attributable burdens, improved lipid management, and wider access to preventive and therapeutic services may have contributed to declining incidence and mortality, although persistent social inequalities may have limited the magnitude of these gains. In Iraq, increasing cardiometabolic exposure, particularly burdens attributable to a high body mass index and elevated fasting plasma glucose levels, may have contributed to the persistence and recent increase in incidence. These effects may be compounded by a high underlying burden of hypertension, rapid population growth, persistent rheumatic heart disease, and health-system constraints affecting early detection, treatment continuity, and long-term risk factor control. These factors should not be interpreted as statistically demonstrated mediators because the present ecological analysis cannot establish individual-level causal pathways. Rather, they represent plausible explanatory mechanisms supported by the observed risk factor trends and the wider literature.

### 4.2. Comparison with Existing Literature

The revised findings are consistent with the broader literature showing that age-standardized cardiovascular mortality has declined in many settings, while the absolute burden remains high because the population size and age structure also change [1,2,3,4,17,20]. The current analysis provides an annual, age-standardized description of the temporal trajectories in the two countries.

Global burden. The reading of the global series, absolute deaths rising while crude and age-standardized rates fall agrees with GBD 2023, which reports a 6.1% rise in total DALYs alongside a 12.6% fall in age-standardized DALY rates between 2010 and 2023 [17], and with Tan et al. [1], who report a 34.3% fall in the global age-standardized CVD mortality rate between 1990 and 2021. Projection work built on GBD 2019 reaches the same structural conclusion for the coming decades [2]. Regional analyses that emphasize improving age-standardized rates are not inconsistent with rising counts; the two statements use different metrics. The regional age-standardized benchmarks used here follow the reference compilation by Mensah et al. [21]. The scale of what remains is not only epidemiological: cardiovascular disease was estimated to cost the 27 European Union countries EUR 282 billion in 2021, equivalent to 11% of European Union health expenditure, of which informal care accounted for 28% [20].

Hungary. The Hungarian pattern is consistent with evidence of persistent cardiovascular inequalities, despite improvements in national-level risk profiles [8,9,10,22]. The finding of a high but slowly improving Hungarian burden, with the decline flattening after approximately 2010, is consistent with Cherla et al. [23], who applied the joint OECD/Eurostat list of preventable and treatable causes of death [22] and found avoidable cardiovascular mortality across the European Union falling by 57% from its 1995 peak to 2020, though with uneven improvement. This is consistent with Roth et al. [4], who noted that age-standardized cardiovascular rates have begun to rise again in some high-income settings after a long period of decline. This is also consistent with Hungarian evidence that risk remains socially concentrated rather than uniformly distributed. Andréka et al. [9] documented a very high prevalence of overweight and physical inactivity across underprivileged settlements, with the sharpest Roma versus non-Roma contrast in smoking (60.3% versus 28.7%), while hypertension and diabetes were somewhat more prevalent among non-Roma settlement residents. Pikó et al. [10] reported a higher estimated 10-year cardiovascular risk in the Hungarian Roma population than in the general population. Both studies were small: 6211 screening participants in the first and 380 general and 347 Roma participants in the second, with the screening sample being approximately three-quarters female. Neither is nationally representative; therefore, they establish the concentration of risk rather than its national magnitude.

Iraq. The Iraqi pattern is consistent with evidence of substantial hypertension, diabetes, smoking, and obesity burdens, as well as reports of health-system disruption and younger clinical presentation of acute coronary syndrome [6,14,24]. The finding of a flat-then-rising incidence and a less favorable metabolic risk trajectory agrees with Merzah [11], who reported that the age-standardized incidence of diabetes and kidney disease increased by 42.9% in Iraq between 2003 and 2021, while cardiovascular disease remained the leading cause of death in both sexes, with age-standardized mortality rising slightly in males and decreasing in females. This finding also agrees with the regional picture described by Soleimani et al. [6], in which countries with a history of war or ongoing conflict have higher cardiovascular death, DALY, and incidence rates than more stable regional neighbors, and with wider evidence on obesity and dysglycemia in the region [24] and the burden attributable to high systolic blood pressure [25] (using GBD 2019 for 1990–2019).

### 4.3. Interpreting the Hungarian Pattern

In Hungary, the prolonged decline in age-standardized CVD mortality and incidence is compatible with cumulative improvements in prevention and treatment, alongside long-standing socioeconomic inequalities in exposure and access [4,8,9,10]. The late-period flattening of several fitted curves suggests that additional reductions may be harder to achieve when the residual risk is concentrated in specific populations.

The age-standardized risk-attributable cardiovascular mortality trajectories declined substantially in Hungary between 1990 and 2023. The observed smoking-attributable mortality rate decreased by 65.5%, from 80.23 to 27.67 per 100,000; the high LDL cholesterol-attributable rate decreased by 58.3%, from 142.13 to 59.21 per 100,000; and the air-pollution-attributable rate decreased by 76.3%, from 119.52 to 28.33 per 100,000. The fitted trajectories declined throughout most of the study period, with varying degrees of late-period flattening, and broadly paralleled the decline in overall age-standardized cardiovascular mortality. These descriptive patterns are consistent with long-term improvements in tobacco exposure, lipid-related risk, and air pollution, although the ecological analysis cannot attribute the overall mortality decline to any individual risk factor. The smoking trajectory is also consistent with the reported global decline in smoking-attributable cardiovascular mortality [26].

Those gains coexist with a persistently high burden, and the resolution is distributional rather than contradictory: population-level improvement in average exposure is compatible with sustained excess outcomes when risk reduction reaches some social groups but not others. Hungarian evidence supports this reading directly [9,10]. The apparent tension between a falling national smoking-attributable burden and very high smoking prevalence in specific subgroups is a difference in metrics, a long-run national attributable trend versus cross-sectional subgroup prevalence, rather than a contradiction. On this reading, Hungary’s excess cardiovascular burden is now driven less by a universal worsening of classic risk factors than by long-standing social stratification in exposure, prevention, and control. The flattening of Hungarian mortality decline after approximately 2010, visible in Figure 2 and Figure 3 and concealed by any single linear slope, deserves attention in its own right.

### 4.4. Interpreting the Iraqi Pattern

In Iraq, the smaller decline in overall age-standardized mortality and incidence was accompanied by less favourable trajectories for selected metabolic risks. Between 1990 and 2023, the observed age-standardized cardiovascular mortality rate attributable to high body-mass index increased by 30.0%, from 60.63 to 78.84 per 100,000, while the rate attributable to high fasting plasma glucose increased by 9.9%, from 61.69 to 67.77 per 100,000. The fitted BMI-attributable trajectory reached a local maximum around 2006.7, declined to a local minimum around 2017.6, and subsequently increased toward 2023. The glucose-attributable trajectory showed a similar pattern, with a fitted maximum around 2006.4 and a minimum around 2018.6 before increasing again. These non-linear trajectories are compatible with an evolving cardiometabolic risk profile and with regional evidence concerning obesity and dysglycaemia [6,24,27]. However, they represent descriptive ecological co-trajectories and should not be interpreted as statistically demonstrated mediators or causal determinants of the overall cardiovascular trend. Hypertension warrants separate consideration. The observed age-standardized cardiovascular mortality rate attributable to high systolic blood pressure in Iraq decreased by 18.4%, from 328.96 per 100,000 in 1990 to 268.36 per 100,000 in 2023. However, the fitted trajectory was non-linear: it reached a local maximum around 1998.7, subsequently declined to a local minimum around 2020.1, and then increased modestly toward 2023. Despite the net decline, the attributable mortality rate remained high throughout the study period. This is consistent with the substantial regional burden attributable to raised blood pressure [26], the high prevalence and limited awareness, treatment, and control of hypertension in the Middle East and North Africa [28], and the frequent occurrence of hypertension among Iraqi patients presenting with acute coronary syndrome [13]. The results should be interpreted alongside measured population-representative exposure data rather than as evidence of a causal relationship between the fitted risk trajectory and overall cardiovascular mortality. National prevalence, treatment, and control estimates are available from the NCD Risk Factor Collaboration [29], with additional Iraq-specific evidence provided by the national STEPS survey [30].

Here, hospital-based Iraqi series are used only to characterize the clinical presentation. Allami [13] conducted a cross-sectional study of 600 patients admitted with acute coronary syndrome to a single teaching hospital in Duhok between 2020 and 2022, reporting smoking in 57.0%, hyperlipidemia in 56.2%, hypertension in 47%, diabetes in 40.7%, and overweight in 29.8%. These are frequencies in a selected high-risk clinical population.

Two further points require careful consideration. First, the change in the physical activity-attributable death rate is small, and its uncertainty interval crosses zero in both countries (Hungary −3.6%, −42.4 to +77.5; Iraq −6.2%, −35.1 to +35.3); therefore, the data do not support characterizing either country by its physical activity level. Second, the estimates show a rising attributable burden from other environmental risks in Iraq, with an interval excluding zero. This is plausible in a setting where urbanization, insecurity, conflict-related disruption, and limited infrastructure act together, and where reduced continuity of care, medication interruption, and chronic stress may amplify the cardiovascular effects of common metabolic risks [6]. The rise in obesity- and glucose-related burden in Iraq should therefore be interpreted not solely as a lifestyle phenomenon but as one element of a broader context of health-system fragility and social disruption.

Finally, the supplementary crude population-scale analysis illustrates why the demographic contribution should not be understated. Iraq’s attributable cardiovascular deaths increased by 116% to 291% in absolute numbers across all risk factors examined, while the corresponding rates ranged between −24% and +64%. Even for high systolic blood pressure, where the per-capita rate fell slightly, the number of attributable deaths increased by 134%. Prevention policy is properly framed in terms of rates because rates describe risk; service planning must be framed in terms of numbers because numbers describe demand. In Iraq, these two framings diverge sharply, and a strategy based solely on the rate would substantially understate what the health system must absorb.

### 4.5. Risk-Factor Trajectories

The selected risk factor trajectories provided a descriptive context for the overall mortality trends. In Hungary, smoking, high LDL cholesterol, and air pollution-attributable CVD mortality declined substantially from 1990 to 2023, broadly paralleling the decline in overall CVD mortality rate. In Iraq, BMI-attributable mortality increased, whereas high fasting plasma glucose and high systolic blood pressure showed more complex patterns. The similarity or divergence of these trajectories does not establish causation because the GBD-attributable burden is modelled within a comparative risk framework and risk pathways overlap [17,27]. In particular, the risk curves should not be interpreted as independent contributions that can be summed or as evidence that a change in a single exposure causes contemporaneous change in overall mortality.

### 4.6. Rheumatic Heart Disease

Rheumatic heart disease remains an important distinguishing component of the Iraqi cardiovascular profile. In 2023, its age-standardized incidence remained far higher in Iraq than in Hungary (43.84 versus 4.57 per 100,000), although mortality rates were low in both countries. The persistent incidence burden is consistent with GBD-based evidence that rheumatic heart disease remains concentrated in lower-sociodemographic-index populations [31]. The present study cannot identify the specific contribution of primary prevention, secondary prophylaxis, diagnostic access, or treatment continuity, but the temporal pattern supports retaining rheumatic heart disease as a distinct component of comparative cardiovascular planning. It marks a genuine difference in disease profile: Hungary’s burden is almost entirely atherosclerotic and degenerative, whereas Iraq carries a mixed profile combining rising cardiometabolic disease with a persistent post-streptococcal component that is, in principle, preventable through primary care access and secondary prophylaxis.

### 4.7. Implications for Prevention

The contrasting patterns indicate that prevention priorities should be adapted to the epidemiological and health-system context of each country. In Hungary, the principal opportunity is not only to reduce average population risk further, but also to address its unequal social distribution. Priorities include targeted tobacco-control interventions, improved detection and sustained control of hypertension, dyslipidemia, and diabetes, and accessible weight-management and physical activity support for disadvantaged populations. Prevention programmes should therefore combine population-wide measures with proportionate targeting of communities in which exposure and access to preventive care remain least favourable.

In Iraq, prevention should focus on the expanding cardiometabolic risk pool while accounting for the rapid growth in absolute service demand. Integrating blood pressure, diabetes, obesity, and lipid screening into primary care, ensuring reliable access to essential medicines, and strengthening continuity of long-term treatment could improve early risk factor control. Renewed population-based surveillance would also be needed to identify priority groups and evaluate progress. In addition, the higher incidence of rheumatic heart disease supports strengthening the timely diagnosis and treatment of streptococcal infection and the delivery of secondary prophylaxis for patients with a history of rheumatic fever. These actions should be accompanied by health-system planning based on both rates and absolute case numbers, because declining or stable per-capita risk may coexist with rapidly increasing demand in a growing population.

### 4.8. Strengths and Limitations

The analysis is primarily descriptive and exploratory; consequently, it characterizes population-level patterns and associations but cannot establish causality, formally identify mediating mechanisms, or determine whether the observed country differences are statistically significant beyond the uncertainty reported for the underlying GBD estimates.

The principal strength is the use of internally consistent GBD 2023 estimates for both countries across the complete annual 1990–2023 series, with age-standardization addressing the major demographic comparability problem. The RCS framework captures non-linear change that would be obscured by a single average annual percentage change, and the FGLS AR (1) specification acknowledges serial dependence in the annual modelled estimates. The study also separates the principal age-standardized analysis from the earlier crude all-age analysis and restricts the risk-factor extension to a small prespecified set. Limitations include the model-based nature of GBD estimates, possible differences in underlying data quality between countries, the ecological level of analysis, and the fact that mortality and incidence are not linked at the individual level. The confidence bands and regression *p*-values should therefore be treated as model-based descriptive quantities rather than population-inference statements based on independent annual observations. The MIR is an ecological proxy and cannot be interpreted as case fatality. The risk-attributable trajectories do not establish causality, and no decomposition into population growth, population ageing, and epidemiological change was performed within the present revision.

### 4.9. Future Research Priorities

Future work should extend the annual age-standardized framework to sex-specific and subnational analyses, incorporate draw-level uncertainty into joint modelling where feasible, and formally decompose changes in absolute cardiovascular burden into demographic and epidemiological components. Additional work linking population-representative risk-factor surveillance with GBD trends would also help distinguish changes in exposure, detection, and treatment from changes arising from the underlying estimation framework.

## 5. Conclusions

Hungary and Iraq carry substantial but structurally different cardiovascular burdens, and age-standardized GBD 2023 estimates show that Hungary and Iraq followed different cardiovascular trajectories between 1990 and 2023. CVD mortality declined by 45.7% in Hungary and 21.1% in Iraq, while CVD incidence declined by 43.9% and 15.4%, respectively. By 2023, Iraq had the higher age-standardized CVD mortality and incidence rates. The annual RCS analysis demonstrates that these trajectories were non-linear and differed by country.

The cause-specific analyses indicate that the pattern was not uniform across cardiovascular diseases, with particularly large reductions in age-standardized stroke and rheumatic heart disease mortality in Hungary and a persistently higher rheumatic heart disease incidence rate in Iraq. The selected risk-factor analyses provide complementary descriptive context: smoking, high LDL cholesterol and air-pollution-attributable CVD mortality declined substantially in Hungary, whereas BMI-attributable burden increased in Iraq and glucose-related burden showed a less favourable trajectory. These ecological co-trajectories do not establish causal effects.

These findings should be interpreted in light of the model-based nature of GBD estimates, serial dependence in annual estimates, and ecological design. They support continued attention to country-specific prevention and health-system priorities while underscoring the need for future analyses using sex-specific, subnational, and draw-level data to investigate the mechanisms underlying divergent cardiovascular trajectories.

## Figures and Tables

**Table 1:** Global Cardiovascular Disease Deaths, Selected Years, 1990–2023.

Year	Deaths (Mean)	95% UI	Crude Death Rate per 100,000	95% UI
1990	13,137,959	12,200,605–14,040,764	246.21	228.64–263.13
2000	14,562,528	13,675,272–15,351,655	238.40	223.88–251.32
2010	15,688,390	14,691,689–16,519,818	225.30	210.99–237.24
2020	17,658,563	16,115,061–18,749,597	224.96	205.29–238.85
2023	19,139,018	17,355,527–20,403,360	237.29	215.17–252.96

Rates are crude and all-age. GBD 2023 cause-specific estimates began in 1990.

**Table 2:** RCS-Predicted Age-Standardized All-CVD Mortality and Incidence Rates (per 100,000) at Selected Years, Hungary and Iraq, 1990–2023.

Measure	Country	1990	2000	2010	2020	2023
Mortality	Hungary	534.7 (522.0–547.7)	447.5 (440.6–454.4)	351.9 (346.3–357.7)	301.2 (296.0–306.5)	291.0 (284.1–298.0)
	Iraq	543.6 (531.5–556.0)	564.0 (557.2–570.9)	507.5 (500.9–514.2)	419.3 (413.5–425.2)	430.2 (420.6–440.0)
Incidence	Hungary	1105.3 (1093.1–1117.6)	1004.8 (995.1–1014.6)	790.3 (782.5–798.1)	622.7 (616.4–629.0)	620.3 (613.5–627.2)
	Iraq	955.6 (945.0–966.2)	968.9 (960.5–977.3)	913.3 (905.2–921.5)	810.7 (803.1–818.4)	808.8 (799.9–817.8)

Values are model-based predictions, with 95% confidence intervals.

**Table 3:** Restricted Cubic Spline Model Tests for Annual Age-Standardized Mortality and Incidence.

Measure	Cause	Overall Year χ^2^ (df)	*p*	Non-Linearity χ^2^ (df)	*p*	Country × Spline χ^2^ (df)	*p*
Mortality	Cardiovascular diseases	293.51 (10)	<0.001	122.87 (8)	<0.001	164.53 (5)	<0.001
Incidence	Cardiovascular diseases	339.43 (10)	<0.001	174.56 (8)	<0.001	244.88 (5)	<0.001
Mortality	Ischaemic heart disease	166.78 (10)	<0.001	68.98 (8)	<0.001	63.75 (5)	<0.001
Incidence	Ischaemic heart disease	371.52 (10)	<0.001	220.80 (8)	<0.001	279.77 (5)	<0.001
Mortality	Stroke	348.54 (10)	<0.001	123.62 (8)	<0.001	237.74 (5)	<0.001
Incidence	Stroke	290.96 (10)	<0.001	119.65 (8)	<0.001	191.56 (5)	<0.001
Mortality	Rheumatic heart disease	232.92 (10)	<0.001	84.03 (8)	<0.001	103.62 (5)	<0.001
Incidence	Rheumatic heart disease	271.10 (10)	<0.001	179.09 (8)	<0.001	229.61 (5)	<0.001

The statistics are likelihood ratio tests from the FGLS models with within-country AR (1) correlation. The *p*-values and confidence bands are model-based descriptive summaries because the annual GBD estimates are serially related posterior estimates.

**Table 4:** Age-Standardized Mortality and Incidence Rates for the Four Prespecified Outcomes in 1990 and 2023.

Measure	Cause	Hungary 1990	Hungary 2023	Change	Iraq 1990	Iraq 2023	Change
Deaths	Cardiovascular diseases	532.96	289.20	−45.7%	544.83	429.66	−21.1%
Deaths	Ischaemic heart disease	255.97	165.37	−35.4%	271.16	230.96	−14.8%
Deaths	Stroke	185.53	56.47	−69.6%	217.11	140.08	−35.5%
Deaths	Rheumatic heart disease	6.82	1.04	−84.8%	4.51	2.19	−51.5%
Incidence	Cardiovascular diseases	1105.34	620.14	−43.9%	955.53	808.65	−15.4%
Incidence	Ischaemic heart disease	580.57	219.54	−62.2%	492.34	369.09	−25.0%
Incidence	Stroke	259.49	115.52	−55.5%	242.61	190.99	−21.3%
Incidence	Rheumatic heart disease	10.54	4.57	−56.6%	45.10	43.84	−2.8%

Rates are per 100,000 population; change is the percentage change from 1990 to 2023.

**Table 5:** Exploratory Annual Age-Standardized Risk-Attributable CVD Mortality Trajectories for Six Prespecified Country–Risk Pairs.

Country	Risk Factor	1990 Rate	2023 Rate	Observed Change	Non-Linearity *p*
Hungary	Smoking	80.23	27.67	−65.5%	<0.001
Hungary	High LDL cholesterol	142.13	59.21	−58.3%	<0.001
Hungary	Air pollution	119.52	28.33	−76.3%	<0.001
Iraq	High body-mass index	60.63	78.84	+30.0%	<0.001
Iraq	High fasting plasma glucose	61.69	67.77	+9.9%	<0.001
Iraq	High systolic blood pressure	328.96	268.36	−18.4%	<0.001

Observed changes are based on 1990 and 2023 GBD estimates; non-linearity *p*-values are from country-specific FGLS AR (1) spline models.

## Data Availability

All data analyzed in this study are publicly available from the GBD Results Tool (https://vizhub.healthdata.org/gbd-results/ (accessed on 30 May 2026)). The data extraction and analytical procedures are described in the Section 2. Additional details are available from the corresponding authors upon reasonable request.

## Supplementary Materials

The following supporting information can be downloaded at: https://www.mdpi.com/article/10.3390/jcdd13100504/s1, The supplementary files contain the complete annual age-standardized GBD 2023 disease dataset; the six selected risk factor CVD mortality series; RCS model results and selected-year predictions; annual fitted curves and turning points; the original crude analyses; source provenance; and the complete statistical syntax. Table S1: Data extraction and analytical specifications; Table S2: Annual age-standardized disease extraction completeness by measure and cause; Table S3: Restricted cubic spline model tests for the principal outcomes; Table S4: Spline-predicted all-CVD age-standardized rates at selected years, 1990–2023; Table S5: Observed age-standardized endpoints and relative changes, 1990–2023; Table S6: Exploratory RCS results for the six prespecified country-risk factor pairs; Table S7: Selected-year RCS predictions for risk-attributable CVD mortality; Table S8: Local extrema of fitted RCS trajectories; Table S9: Age-standardized mortality-to-incidence ratios by cause; Table S10: Original global crude cardiovascular context, selected years 1990–2023; Table S11: Original crude all-age cardiovascular mortality rates by cause, 1990–2023; Table S12: Original crude all-age cardiovascular incidence rates by cause, 1990–2023; Table S13. Original crude five-year period means for cardiovascular mortality and incidence, 1990–2023; Table S14: Original crude cardiovascular temporal rate of change, 1990–1994 to 2020–2023; Table S15: Original crude risk-attributable cardiovascular death and DALY rate changes, 1990–2023; Table S16: Original crude population-scale risk burden: attributable deaths versus attributable rates, 1990–2023; Table S17: Original crude mortality-to-incidence ratios by cause, 1990–2023; Figure S1: Crude, all-age cardiovascular mortality (A) and incidence (B) per 100,000 population, Hungary and Iraq, period means 1990–2023, with 95% uncertainty intervals shaded; Figure S2: Solid lines with filled markers denote mortality, and dashed lines with open markers denote incidence; Figure S3: Relative changes in cardiovascular death rates attributable to major modifiable risk factors in Hungary and Iraq between 1990 and 2023, with 95% uncertainty intervals; Figure S4: Relative change in attributable cardiovascular deaths, 1990–2023, shown as change in rate (open circles) and change in number (filled circles) for each risk factor. (A) Hungary, where the population fell by 7.4%, and the two measures nearly coincide. (B) Iraq, where the population rose by 138.2%, and the two diverge by 105–227 percentage points; Figure S5: Mortality-to-incidence ratio by cause in Hungary and Iraq on a logarithmic scale.

## Abbreviations

Abbreviations used in this manuscript are as follows:

ASMR	Age-standardized mortality rate
CVD	Cardiovascular disease
DALY	Disability-adjusted life year
ESP	European Standard Population
GBD	Global Burden of Disease
IHD	Ischaemic heart disease
IHME	Institute for Health Metrics and Evaluation
MIR	Mortality-to-incidence ratio
NAME	North Africa and Middle East
UI	Uncertainty interval
YLD	Year lived with disability
YLL	Year of life lost

## References

[1] Tan S.C.W., Zheng B.-B., Tang M.-L., Chu H., Zhao Y.-T., Weng C. (2025). Global burden of cardiovascular diseases and its risk factors, 1990–2021: A systematic analysis for the Global Burden of Disease Study 2021. QJM Int. J. Med..

[2] Chong B., Jayabaskaran J., Jauhari S.M., Chan S.P., Goh R., Kueh M.T.W., Li H., Chin Y.H., Kong G., Anand V.V. (2025). Global burden of cardiovascular diseases: Projections from 2025 to 2050. Eur. J. Prev. Cardiol..

[3] Naghavi M., Duncan B.B., Schmidt M.I., Mattiello R., Murray C.J., GBD 2023 Causes of Death Collaborators (2025). Global burden of 292 causes of death in 204 countries and territories and 660 subnational locations, 1990–2023: A systematic analysis for the Global Burden of Disease Study 2023. Lancet.

[4] Roth G.A., Mensah G.A., Johnson C.O., Addolorato G., Ammirati E., Baddour L.M., Barengo N.C., Beaton A.Z., Benjamin E.J., Benziger C.P. (2020). Global burden of cardiovascular diseases and risk factors, 1990–2019: Update from the GBD 2019 study. J. Am. Coll. Cardiol..

[5] World Health Organization (2025). Cardiovascular Diseases (CVDs). Fact Sheet. https://www.who.int/news-room/fact-sheets/detail/cardiovascular-diseases-(cvds).

[6] Soleimani H., Nasrollahizadeh A., Nasrollahizadeh A., Razeghian I., Molaei M.M., Hakim D., Nasir K., Al-Kindi S., Hosseini K. (2024). Cardiovascular disease burden in the North Africa and Middle East region: An analysis of the Global Burden of Disease study 1990–2021. BMC Cardiovasc. Disord..

[7] Yusuf S., Joseph P., Rangarajan S., Islam S., Mente A., Hystad P., Brauer M., Kutty V.R., Gupta R., Wielgosz A. (2020). Modifiable risk factors, cardiovascular disease, and mortality in 155,722 individuals from 21 high-income, middle-income, and low-income countries (PURE): A prospective cohort study. Lancet.

[8] OECD, European Observatory on Health Systems and Policies (2023). Hungary: Country Health Profile 2023.

[9] Andréka L., Csenteri-Bárdos O., Szőllősi G.J., Andréka P., Jancsó Z., Vajer P. (2024). Cardiovascular risk factors among Roma and non-Roma populations in underprivileged settlements. BMC Prim. Care.

[10] Pikó P., Kósa Z., Sándor J., Ádány R. (2021). Comparative risk assessment for the development of cardiovascular diseases in the Hungarian general and Roma population. Sci. Rep..

[11] Merzah M. (2025). Trends in incidence, prevalence, and mortality of non-communicable diseases in Iraq (2003–2021). BMC Public Health.

[12] Allami M. (2024). A cross-sectional study on the epidemiology and risk factors of acute coronary syndrome in northern Iraq. Cureus.

[13] Alhuneafat L., Al Ta’ani O., Jabri A., Tarawneh T., ElHamdan A., Naser A., Al-Bitar F., Alrifai N., Ghanem F., Alaswad K. (2024). Cardiovascular disease burden in the Middle East and North Africa region. Curr. Probl. Cardiol..

[14] Vaduganathan M., Mensah G.A., Turco J.V., Fuster V., Roth G.A. (2022). The global burden of cardiovascular diseases and risk: A compass for future health. J. Am. Coll. Cardiol..

[15] Global Burden of Disease Collaborative Network, Global Burden of Disease Study 2023 (GBD 2023) (2024). Results.

[16] GBD 2023 Diseases, Injuries, and Risk Factors Collaborators (2025). Burden of 375 diseases and injuries, risk-attributable burden of 88 risk factors, and healthy life expectancy in 204 countries and territories, including 660 subnational locations, 1990–2023: A systematic analysis for the Global Burden of Disease Study 2023. Lancet.

[17] United Nations, Department of Economic and Social Affairs, Population Division (2024). World Population Prospects 2024.

[18] World Heart Federation (2025). World Heart Observatory: Hungary. https://world-heart-federation.org/world-heart-observatory/countries/hungary/.

[19] Eurostat (2024). Cardiovascular Diseases Statistics.

[20] Luengo-Fernandez R., Walli-Attaei M., Gray A., Torbica A., Maggioni A.P., Huculeci R., Bairami F., Aboyans V., Timmis A.D., Vardas P. (2023). Economic burden of cardiovascular diseases in the European Union: A population-based cost study. Eur. Heart J..

[21] Mensah G.A., Fuster V., Murray C.J.L., Roth G.A., Global Burden of Cardiovascular Diseases and Risks Collaborators (2023). Global burden of cardiovascular diseases and risks, 1990–2022. J. Am. Coll. Cardiol..

[22] OECD, Eurostat (2022). Avoidable Mortality: OECD/Eurostat Lists of Preventable and Treatable Causes of Death.

[23] Cherla A., Kyriopoulos I., Pearcy P., Tsangalidou Z., Hajrulahovic H., Theodorakis P., Andersson C.E., Mehra M.R., Mossialos E. (2024). Trends in avoidable mortality from cardiovascular diseases in the European Union, 1995–2020: A retrospective secondary data analysis. Lancet Reg. Health Eur..

[24] El-Kebbi I.M., Bidikian N.H., Hneiny L., Nasrallah M.P. (2021). Epidemiology of type 2 diabetes in the Middle East and North Africa: Challenges and call for action. World J. Diabetes.

[25] Safiri S., Mousavi S.E., Motlagh Asghari K., Nejadghaderi S.A., Aletaha R., Sullman M.J.M., Singh K., Kolahi A.-A., Beyranvand M.R. (2024). Burden of diseases due to high systolic blood pressure in the Middle East and North Africa region from 1990 to 2019. Sci. Rep..

[26] Zhu S., Gao J., Zhang L., Dong W., Shi W., Guo H., Zhang X., Hua T., Yang M. (2025). Global, regional, and national cardiovascular disease burden attributable to smoking from 1990 to 2021: Findings from the GBD 2021 study. Tob. Induc. Dis..

[27] Huang P., Guo Y., Ren G., Guo L., Wang Y., Liu Y., Zhang Z., Ma X. (2026). Global trends of cardiovascular disease burden attributable to high body mass index from 1990 to 2021 and projections to 2035. Front. Cardiovasc. Med..

[28] Balouchi A., Rafsanjani M.H.A.P., Al-Mutawaa K., Naderifar M., Rafiemanesh H., Ebadi A., Ghezeljeh T.N., Shahraki-Mohammadi A., Al-Mawali A. (2022). Hypertension and pre-hypertension in Middle East and North Africa (MENA): A meta-analysis of prevalence, awareness, treatment, and control. Curr. Probl. Cardiol..

[29] NCD Risk Factor Collaboration (NCD-RisC) (2021). Worldwide trends in hypertension prevalence and progress in treatment and control from 1990 to 2019: A pooled analysis of 1201 population-representative studies with 104 million participants. Lancet.

[30] World Health Organization, Ministry of Health of Iraq (2015). Iraq STEPS Noncommunicable Disease Risk Factors Survey 2015.

[31] Zhang J., Ren T., Zhao Q. (2025). Rheumatic heart disease burden from 1990 to 2021: An updated analysis based on the Global Burden of Disease Study 2021. Front. Public Health.

