# Supplementary material for: Divergent Cardiovascular Disease Burden in Hungary and Iraq: A Comparative Analysis of Global Burden of Disease Estimates
Source: J Cardiovasc Dev Dis. 2026 Oct 8;13(10):504. doi: 10.3390/jcdd13100504 (PMC13705221; doi:10.3390/jcdd13100504)
Supplement: Supplementary file 1 [file jcdd-13-00504-s001.zip › jcdd-4566266-supplementary.pdf]

Supplementary Materials

Supplementary analytical dataset, RCS model outputs, selected risk factor trajectories, and retained crude analyses. This supplementary file documents the annual GBD 2023 extraction and analyses used in the revised manuscript. The principal country comparison used age-standardized annual rates for 1990–2023. Earlier crude all-age analyses were retained only as supplementary descriptive/sensitivity materials. The accompanying Excel workbook contains complete annual records and machine-readable model outputs.

S1. Data sources and analytical specifications

Table S1. Data extraction and analytical specifications

Item	Specification
**Locations**	Hungary; Iraq
**Years**	Every calendar year, 1990–2023
**Sex**	Both
**Age**	Age-standardized
**Metric**	Rate per 100,000 population
**Disease measures**	Deaths; Incidence
**Principal RCS causes**	Cardiovascular diseases; Ischaemic heart disease; Stroke; Rheumatic heart disease
**Level-3 extraction**	13 mortality cause-series and 10 incidence cause-series; incidence unavailable for aortic aneurysm, hypertensive heart disease, and other cardiovascular and circulatory diseases
**RCS specification**	log(rate) = country + RCS (year) + country×RCS(year)
**Knots**	5th, 35th, 65th, 95th percentiles: 1991.65, 2001.55, 2011.45, 2021.35
**Serial dependence**	FGLS with within-country AR (1) covariance
**Selected risk analysis**	Six prespecified country-risk pairs; annual age-standardized attributable CVD mortality
**Risk index**	Fitted risk trajectories indexed to 1990 = 100; no causal mediation inference; no risk summation

Note: The specifications refer to the principal annual GBD 2023 extraction used for the revised analysis. Rates are age-standardized for both sexes and expressed per 100,000 population. Level-3 extraction includes all available annual cause series; incidence was unavailable for the three causes identified in the table.

S2. Annual age-standardized disease data completeness

Table S2. Annual age-standardized disease extraction completeness by measure and cause

Measure	Cause	Rows	Years	Countries	Duplicates
**Deaths**	Aortic aneurysm	68	34	2	0
**Deaths**	Atrial fibrillation and flutter	68	34	2	0
**Deaths**	Cardiomyopathy and myocarditis	68	34	2	0
**Deaths**	Cardiovascular diseases	68	34	2	0
**Deaths**	Endocarditis	68	34	2	0
**Deaths**	Hypertensive heart disease	68	34	2	0
**Deaths**	Ischaemic heart disease	68	34	2	0
**Deaths**	Lower-extremity peripheral arterial disease	68	34	2	0
**Deaths**	Non-rheumatic valvular heart disease	68	34	2	0
**Deaths**	Other cardiovascular and circulatory diseases	68	34	2	0

**Deaths**	Pulmonary arterial hypertension	68	34	2	0
**Deaths**	Rheumatic heart disease	68	34	2	0
**Deaths**	Stroke	68	34	2	0
**Incidence**	Atrial fibrillation and flutter	68	34	2	0
**Incidence**	Cardiomyopathy and myocarditis	68	34	2	0
**Incidence**	Cardiovascular diseases	68	34	2	0
**Incidence**	Endocarditis	68	34	2	0
**Incidence**	Ischaemic heart disease	68	34	2	0
**Incidence**	Lower-extremity peripheral arterial disease	68	34	2	0
**Incidence**	Non-rheumatic valvular heart disease	68	34	2	0
**Incidence**	Pulmonary arterial hypertension	68	34	2	0
**Incidence**	Rheumatic heart disease	68	34	2	0
**Incidence**	Stroke	68	34	2	0

Note: Each complete cause series contains 34 annual observations for each of the two countries (68 rows). A duplicate count of zero indicates no duplicate country-year records in the supplied extraction.

S3. Principal RCS model results

Table S3. Restricted cubic spline model tests for the principal outcomes

Outcome	Cause	N	Overall-year χ^2 (df)	p	Non-linearity χ^2 (df)	p	Country×spline χ^2 (df)	p	ρ Hungary	ρ Iraq
**Mortality**	Cardiovascular diseases	68	293.508 (10)	3.66e-57	122.867 (8)	8.48e-23	164.528 (5)	1.07e-33	0.578	0.356
**Incidence**	Cardiovascular diseases	68	339.432 (10)	6.95e-67	174.564 (8)	1.42e-33	244.882 (5)	6.89e-51	0.863	0.767
**Mortality**	Ischaemic heart disease	68	166.783 (10)	1.29e-30	68.985 (8)	7.82e-12	63.751 (5)	2.03e-12	0.735	0.487
**Incidence**	Ischaemic heart disease	68	371.523 (10)	1.07e-73	220.798 (8)	2.61e-43	279.766 (5)	2.23e-58	0.872	0.634
**Mortality**	Stroke	68	348.536 (10)	8.15e-69	123.618 (8)	5.93e-23	237.742 (5)	2.34e-49	0.504	0.179
**Incidence**	Stroke	68	290.955 (10)	1.27e-56	119.646 (8)	3.92e-22	191.564 (5)	1.81e-39	0.839	0.809
**Mortality**	Rheumatic heart disease	68	232.924 (10)	2.09e-44	84.034 (8)	7.51e-15	103.620 (5)	9.11e-21	0.449	0.796
**Incidence**	Rheumatic heart disease	68	271.096 (10)	1.96e-52	179.090 (8)	1.6e-34	229.611 (5)	1.3e-47	0.884	0.717

Statistics are likelihood-ratio (χ^2) tests from FGLS models with within-country AR(1) covariance. P-values and confidence bands are model-based summaries because annual GBD estimates are serially related posterior estimates rather than independent observations.

S4. predicted age-standardized all-CVD rates at selected years

Table S4. Spline-predicted all-CVD age-standardized rates at selected years, 1990–2023

Measure	Country	Year	Predicted rate	Lower 95% CI	Upper 95% CI
**Mortality**	Hungary	1990	534.7	522.0	547.7
**Mortality**	Hungary	2000	447.5	440.6	454.4
**Mortality**	Hungary	2010	351.9	346.3	357.7
**Mortality**	Hungary	2020	301.2	296.0	306.5
**Mortality**	Hungary	2023	291.0	284.1	298.0
**Mortality**	Iraq	1990	543.6	531.5	556.0
**Mortality**	Iraq	2000	564.0	557.2	570.9
**Mortality**	Iraq	2010	507.5	500.9	514.2
**Mortality**	Iraq	2020	419.3	413.5	425.2
**Mortality**	Iraq	2023	430.2	420.6	440.0
**Incidence**	Hungary	1990	1105.3	1093.1	1117.6
**Incidence**	Hungary	2000	1004.8	995.1	1014.6
**Incidence**	Hungary	2010	790.3	782.5	798.1
**Incidence**	Hungary	2020	622.7	616.4	629.0
**Incidence**	Hungary	2023	620.3	613.5	627.2
**Incidence**	Iraq	1990	955.6	945.0	966.2
**Incidence**	Iraq	2000	968.9	960.5	977.3
**Incidence**	Iraq	2010	913.3	905.2	921.5
**Incidence**	Iraq	2020	810.7	803.1	818.4
**Incidence**	Iraq	2023	808.8	799.9	817.8

Note: Rates are RCS model predictions on the natural logarithm scale, back-transformed to the rate scale. The 95% confidence intervals were model-based and distinct from the 95% uncertainty intervals supplied by the GBD for the observed estimates.

S5. Observed age-standardized endpoints and relative changes

Table S5. Observed age-standardized endpoints and relative changes, 1990–2023.

Measure	Cause	Country	1990	2023	Relative change %
**Deaths**	Cardiovascular diseases	Hungary	532.96	289.20	-45.7
**Deaths**	Rheumatic heart disease	Hungary	6.82	1.04	-84.8
**Deaths**	Stroke	Hungary	185.53	56.47	-69.6
**Deaths**	Cardiovascular diseases	Iraq	544.83	429.66	-21.1
**Deaths**	Rheumatic heart disease	Iraq	4.51	2.19	-51.5
**Deaths**	Stroke	Iraq	217.11	140.08	-35.5
**Incidence**	Cardiovascular diseases	Hungary	1105.34	620.14	-43.9
**Incidence**	Rheumatic heart disease	Hungary	10.54	4.57	-56.6
**Incidence**	Stroke	Hungary	259.49	115.52	-55.5
**Incidence**	Cardiovascular diseases	Iraq	955.53	808.65	-15.4
**Incidence**	Rheumatic heart disease	Iraq	45.10	43.84	-2.8
**Incidence**	Stroke	Iraq	242.61	190.99	-21.3

Note: Values are observed annual GBD 2023 age-standardised estimates at 1990 and 2023. The relative change was calculated as 100 × (2023 estimate − 1990 estimate) / 1990 estimate.

S6. Exploratory RCS analysis of prespecified risk factors

Table S6. Exploratory RCS results for the six prespecified country-risk factor pairs

Country	Risk factor	N	AR (1) ρ	Overall-year χ^2	Overall p	Non-linearity χ^2	Non-linearity p	1990	2023	Change %
**Hungary**	Smoking	34	0.631	165.79	5.77e-34	32.68	1.39e-06	80.23	27.67	-65.5
**Hungary**	High LDL cholesterol	34	0.753	119.76	3.53e-24	21.08	0.000306	142.13	59.21	-58.3
**Hungary**	Air pollution	34	0.558	153.97	1.91e-31	40.53	3.37e-08	119.52	28.33	-76.3
**Iraq**	High body-mass index	34	0.455	117.78	9.28e-24	97.56	3.25e-20	60.63	78.84	30.0
**Iraq**	High fasting plasma glucose	34	0.208	83.46	1.58e-16	82.30	5.66e-17	61.69	67.77	9.9
**Iraq**	High systolic blood pressure	34	0.317	155.84	7.62e-32	105.55	6.46e-22	328.96	268.36	-18.4

Note: These models are six prespecified country-risk analyses. The tests are from country-specific FGLS models with AR(1) covariance; they are exploratory and descriptive and do not constitute causal mediation analyses.

S7. Selected-year predictions for risk-attributable CVD mortality

Table S7. Selected-year RCS predictions for risk-attributable CVD mortality

Country	Risk factor	Year	Predicted rate	Lower 95% CI	Upper 95% CI
**Hungary**	Smoking	1990	80.45	77.61	83.40
**Hungary**	Smoking	2000	61.90	60.41	63.42
**Hungary**	Smoking	2010	41.04	40.00	42.10
**Hungary**	Smoking	2020	29.01	28.23	29.81
**Hungary**	Smoking	2023	27.90	26.91	28.92
**Hungary**	High LDL cholesterol	1990	142.40	135.18	150.01
**Hungary**	High LDL cholesterol	2000	119.85	115.14	124.76
**Hungary**	High LDL cholesterol	2010	83.72	80.31	87.28
**Hungary**	High LDL cholesterol	2020	61.01	58.40	63.73
**Hungary**	High LDL cholesterol	2023	59.53	56.51	62.71
**Hungary**	Air pollution	1990	120.06	113.38	127.14
**Hungary**	Air pollution	2000	90.33	87.13	93.64
**Hungary**	Air pollution	2010	62.04	59.72	64.46
**Hungary**	Air pollution	2020	33.61	32.27	35.01
**Hungary**	Air pollution	2023	28.66	27.07	30.35
**Iraq**	High body-mass index	1990	60.60	59.56	61.67
**Iraq**	High body-mass index	2000	73.67	72.94	74.42
**Iraq**	High body-mass index	2010	76.56	75.74	77.39
**Iraq**	High body-mass index	2020	72.87	72.03	73.71

**Iraq**	High body-mass index	2023	79.10	77.74	80.49
**Iraq**	High fasting plasma glucose	1990	61.32	60.29	62.36
**Iraq**	High fasting plasma glucose	2000	68.10	67.54	68.67
**Iraq**	High fasting plasma glucose	2010	69.03	68.41	69.66
**Iraq**	High fasting plasma glucose	2020	64.42	63.80	65.04
**Iraq**	High fasting plasma glucose	2023	67.60	66.46	68.75
**Iraq**	High systolic blood pressure	1990	328.18	323.22	333.22
**Iraq**	High systolic blood pressure	2000	345.85	343.09	348.63
**Iraq**	High systolic blood pressure	2010	314.07	311.37	316.79
**Iraq**	High systolic blood pressure	2020	260.81	258.42	263.23
**Iraq**	High systolic blood pressure	2023	268.67	264.61	272.79

Note: Values are spline-predicted attributable CVD mortality rates. The 95% confidence intervals are model-based intervals for the fitted RCS trajectories and are not the original GBD 95% uncertainty intervals.

S8. Turning points in fitted RCS trajectories

Table S8. Local extrema of fitted RCS trajectories

**Analysis**	**Outcome/Risk**	**Country**	**Year**	**Type**
**Principal RCS**	Cardiovascular diseases	Iraq	1999.01	maximum
**Principal RCS**	Cardiovascular diseases	Iraq	2020.23	minimum
**Principal RCS**	Cardiovascular diseases	Hungary	2021.74	minimum
**Principal RCS**	Cardiovascular diseases	Iraq	1999.60	maximum
**Principal RCS**	Cardiovascular diseases	Iraq	2021.77	minimum
**Principal RCS**	Ischaemic heart disease	Hungary	1992.45	maximum
**Principal RCS**	Ischaemic heart disease	Iraq	1993.08	minimum
**Principal RCS**	Ischaemic heart disease	Iraq	2002.83	maximum
**Principal RCS**	Ischaemic heart disease	Iraq	2019.40	minimum
**Principal RCS**	Ischaemic heart disease	Iraq	1991.23	minimum
**Principal RCS**	Ischaemic heart disease	Iraq	2001.03	maximum
**Principal RCS**	Stroke	Iraq	1994.81	maximum
**Principal RCS**	Stroke	Hungary	2020.35	minimum
**Principal RCS**	Stroke	Iraq	1992.76	maximum
**Principal RCS**	Stroke	Iraq	2020.00	minimum
**Principal RCS**	Rheumatic heart disease	Hungary	1990.14	maximum
**Principal RCS**	Rheumatic heart disease	Iraq	1994.30	maximum
**Principal RCS**	Rheumatic heart disease	Iraq	2021.94	minimum
**Principal RCS**	Rheumatic heart disease	Hungary	2013.40	minimum
**Principal RCS**	Rheumatic heart disease	Hungary	2021.68	maximum
**Principal RCS**	Rheumatic heart disease	Iraq	1995.17	maximum
**Principal RCS**	Rheumatic heart disease	Iraq	2019.45	minimum
**Risk RCS**	High body-mass index	Iraq	2006.70	maximum
**Risk RCS**	High body-mass index	Iraq	2017.59	minimum
**Risk RCS**	High fasting plasma glucose	Iraq	2006.44	maximum
**Risk RCS**	High fasting plasma glucose	Iraq	2018.62	minimum

**Risk RCS**	High systolic blood pressure	Iraq	1998.72	maximum
**Risk RCS**	High systolic blood pressure	Iraq	2020.10	minimum

Note: Turning points are the numerical local maxima or minima of the fitted spline curves. They are descriptive features of the modelled trajectories and should not be interpreted as causal or epidemiological breakpoints.

Turning points are numerical local maxima/minima of the fitted curves and are descriptive features of the modelled trajectories, not causal or epidemiological breakpoints.

S9. Age-standardized mortality-to-incidence ratios

Table S9. Age-standardized mortality-to-incidence ratios by cause

Cause	Hungary MIR	Iraq MIR
**Cardiovascular diseases**	0.459	0.559
**Ischaemic heart disease**	0.533	0.560
**Stroke**	0.608	0.873
**Rheumatic heart disease**	0.453	0.079
**Atrial fibrillation and flutter**	0.069	0.100
**Cardiomyopathy and myocarditis**	1.361	0.683
**Endocarditis**	0.065	0.042
**Lower-extremity peripheral arterial disease**	0.035	0.001
**Non-rheumatic valvular heart disease**	0.100	0.200
**Pulmonary arterial hypertension**	0.372	0.373

Note: The mortality-to-incidence ratio (MIR) is the mean age-standardized mortality rate divided by the mean age-standardized incidence rate from 1990 to 2023. The MIR is an ecological proxy and is not a case-fatality probability, particularly when incidence and mortality are separated by long time intervals.

S10. Original global crude context

Table S10. Original global crude cardiovascular context, selected years 1990–2023.

Year	Deaths (mean)	95% UI	Crude death rate per 100,000	95% UI
**1990**	13,137,959	12,200,605–14,040,764	246.21	228.64–263.13
**2000**	14,562,528	13,675,272–15,351,655	238.40	223.88–251.32
**2010**	15,688,390	14,691,689–16,519,818	225.30	210.99–237.24
**2020**	17,658,563	16,115,061–18,749,597	224.96	205.29–238.85
**2023**	19,139,018	17,355,527–20,403,360	237.29	215.17–252.96

Note: Global estimates are retained solely as descriptive context for the distinction between absolute burden and population-level rates. They were not included in the Hungary–Iraq comparative analyses. Rates are crude and all ages.

S11. Original crude cardiovascular mortality by cause

Table S11. Original crude all-age cardiovascular mortality rates by cause, 1990–2023

Cause	Hungary means	Hungary median	Hungary SD	Iraq means	Iraq median	Iraq SD	Iraq ÷ Hungary
**Cardiovascular diseases (all)**	666.20	651.47	38.50	199.33	201.57	13.34	0.30
**Ischaemic heart disease**	351.68	355.98	17.64	98.24	98.65	3.85	0.28
**Stroke**	194.31	199.24	54.11	77.24	75.51	11.41	0.40
**Hypertensive heart disease**	45.70	38.68	14.79	12.40	12.63	0.63	0.27

**Cardiomyopathy and myocarditis**	25.11	23.26	5.34	3.85	3.77	0.26	0.15
**Other cardiovascular diseases**	13.30	13.50	5.75	3.20	3.16	0.30	0.24
**Non-rheumatic valvular heart disease**	9.19	8.90	3.71	0.59	0.59	0.11	0.06
**Lower-extremity peripheral arterial disease**	7.23	7.91	1.76	0.02	0.02	0.01	0.003
**Atrial fibrillation and flutter**	6.50	6.18	1.59	1.02	0.98	0.18	0.16
**Rheumatic heart disease**	6.25	4.17	4.14	2.20	2.29	0.54	0.35
**Aortic aneurysm**	5.19	5.38	0.87	0.16	0.16	0.04	0.03
**Endocarditis**	1.41	1.39	0.62	0.24	0.23	0.01	0.17
**Pulmonary arterial hypertension**	0.30	0.29	0.08	0.16	0.17	0.03	0.53

Note: These are the original crude all-age mortality results retained for supplementary transparency. Means, medians, and SDs summarize annual temporal variation; the SD is not an uncertainty interval.

Values are means, medians, and standard deviations of annual crude rates across 1990–2023. The standard deviation describes the variation in annual rates over time, not the estimation uncertainty; uncertainty intervals for the period means are reported.

Crude all-age cardiovascular mortality averaged 666.2 per 100,000 in Hungary and 199.3 per 100,000 in Iraq across 1990–2023, placing Iraq at 0.30 of the Hungarian rates. Crude incidence averaged 1399.6 and 447.7 per 100,000, respectively, placing Iraq at 0.32 of the Hungarian rates. Ischemic heart disease and stroke were dominant in both countries.

The single substantive exception runs in the opposite direction: the incidence of rheumatic heart disease is 5.5 times higher in Iraq than in Hungary (52.84 versus 9.56 per 100,000), the only cause for which Iraq exceeds Hungary on either measure.

These crude differences should not be interpreted as differences in underlying cardiovascular risk. As set out in Section 2.3, Hungary's median age is more than twice that of Iraq, and cardiovascular event rates increase steeply with age; a substantial share of the 3.3-fold crude gap therefore reflects differences in demography rather than in exposure, prevention, or treatment.

S12. Original crude cardiovascular incidence by cause

Table S12. Original crude all-age cardiovascular incidence rates by cause, 1990–2023

**Cause**	**Hungary means**	**Hungary SD**	**Hungary range**	**Iraq means**	**Iraq SD**	**Iraq range**	**Iraq ÷ Hungary**
**Cardiovascular diseases (all)**	1399.63	129.21	1213.14–1555.92	447.72	10.26	437.54–482.79	0.32
**Ischaemic heart disease**	656.98	137.56	445.98–813.49	195.30	3.90	187.97–204.55	0.30
**Stroke**	278.53	50.26	215.51–348.01	110.82	5.75	101.09–120.89	0.40
**Lower-extremity peripheral arterial disease**	216.29	26.53	170.99–255.63	49.94	9.02	41.34–69.12	0.23
**Atrial fibrillation and flutter**	102.99	7.27	94.84–118.03	17.24	1.96	15.37–21.93	0.17
**Non-rheumatic valvular heart disease**	99.47	19.68	68.07–125.46	3.37	0.47	2.88–4.48	0.03
**Endocarditis**	23.54	7.47	11.10–32.34	8.80	0.88	7.63–10.21	0.37
**Cardiomyopathy and myocarditis**	11.60	0.13	11.32–11.72	9.03	0.23	8.62–9.50	0.78

**Rheumatic heart disease**	9.56	2.42	7.36–14.36	52.84	2.01	49.82–55.58	5.53
**Pulmonary arterial hypertension**	0.67	0.06	0.50–0.73	0.40	0.03	0.35–0.45	0.60

Note: These are the original crude, all-age incidence results retained for supplementary transparency. The reported range describes the observed annual range from 1990 to 2023 and is not a GBD uncertainty interval.

S13. Original five-year crude period summaries

Table S13. Original crude five-year period means for cardiovascular mortality and incidence, 1990–2023

Period	Mortality, Hungary (95% UI) (bounds)	Mortality, Iraq (95% UI) (bounds)	Incidence, Hungary (95% UI) (bounds)	Incidence, Iraq (95% UI) (bounds)
**1990–1994**	709.99 (680.40–726.34)	209.50 (181.94–233.50)	1533.78 (1440.29–1640.78)	448.66 (417.90–485.29)
**1995–1999**	682.93 (653.53–701.09)	204.61 (180.91–227.93)	1539.39 (1439.36–1639.17)	441.62 (411.84–473.07)
**2000–2004**	645.09 (613.28–662.03)	199.41 (179.30–221.15)	1489.32 (1389.29–1603.84)	440.14 (409.82–470.85)
**2005–2009**	638.12 (603.07–657.17)	197.98 (178.89–217.83)	1427.97 (1330.69–1545.80)	445.26 (416.81–475.48)
**2010–2014**	623.47 (582.63–645.61)	186.67 (167.77–204.00)	1321.09 (1225.15–1433.09)	446.84 (418.19–478.45)
**2015–2019**	630.44 (586.50–653.07)	177.10 (157.93–195.84)	1231.69 (1140.42–1342.59)	446.07 (416.15–479.15)
**2020–2023**	631.34 (589.07–653.61)	186.15 (159.30–212.12)	1217.79 (1130.72–1339.00)	469.90 (436.72–506.59)

Note: Period values are the arithmetic means of the annual crude rates within each interval. The accompanying bounds are the averages of the annual GBD uncertainty limits and should not be interpreted as formally derived uncertainty intervals for the period means.

Values are period means of annual crude rates, with 95% uncertainty intervals. Intervals reflect estimation uncertainty in the underlying modelled estimates, not sampling uncertainty.

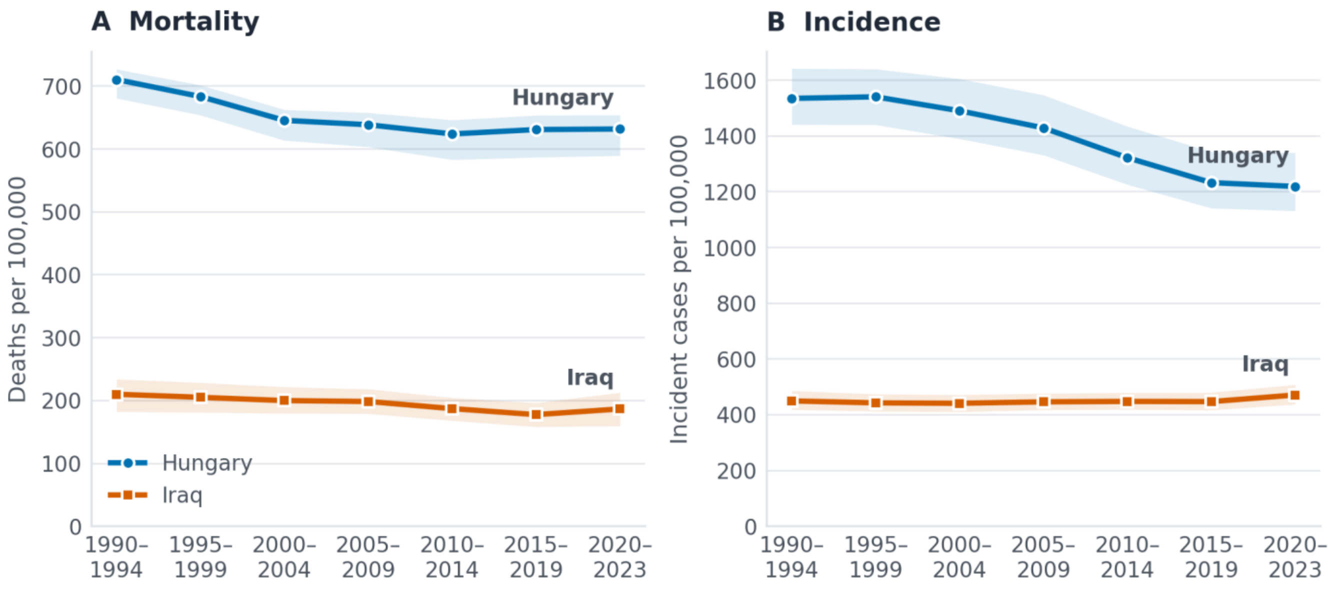

Supplementary Figure S1. Crude, all-age cardiovascular mortality (A) and incidence (B) per 100,000 population, Hungary and Iraq, period means 1990–2023, with 95% uncertainty intervals shaded. Rates are crude and all-age, not age-standardized.

Period means and their 95% UIs are given in Supplementary Table S13 and plotted in Supplementary Figure S1. In Hungary, crude mortality fell from 709.99 per 100,000 in 1990–1994 to 631.34 in 2020–2023; in Iraq, it fell from 209.50 to 186.15. Hungarian incidence declined steadily, from 1533.78 to 1217.79, whereas Iraqi incidence was broadly flat until 2015–2019 and then rose, reaching 469.90 in 2020–2023, its highest value in the series.

S14. Original crude temporal rate of change

Table S14. Original crude cardiovascular temporal rate of change, 1990–1994 to 2020–2023

Outcome	Country	First period	Last period	Absolute change	Relative change	Mean annual change
**Mortality**	Hungary	709.99	631.34	−78.65	−11.08%	−0.396%/yr
**Mortality**	Iraq	209.50	186.15	−23.35	−11.15%	−0.513%/yr
**Incidence**	Hungary	1533.78	1217.79	−315.99	−20.60%	−0.904%/yr
**Incidence**	Iraq	448.66	469.90	+21.24	+4.73%	+0.124%/yr

Note: The mean annual change is the log-linear slope across the seven period midpoints. This is a retained supplementary summary and is not the basis for the principal annual RCS analysis.

Rates per 100,000 population. The mean annual change is the log-linear slope across period midpoints, expressed as a percentage. Absolute changes are shown alongside relative changes to make the scale dependence explicit.

Expresses the same series on the relative scale. Between 1990–1994 and 2020–2023, crude cardiovascular mortality fell by 11.08% in Hungary and by 11.15% in Iraq. The mean annual change was −0.396% per year in Hungary and −0.513% per year in Iraq. On this scale, the proportional declines were numerically very similar, and if anything, Iraq improved marginally faster. Hungary’s larger absolute decrement (−78.65 versus −23.35 per 100,000) reflects its 3.4-fold higher starting level, not a steeper trajectory.

The incidence contrast is of a different order and survives at every scale. The Hungarian crude incidence fell by 20.60% (−0.904% per year), whereas the Iraqi crude incidence rose by 4.73% (+0.124% per year), a divergence of approximately 25 percentage points over the period, as shown in Supplementary Figure S2.

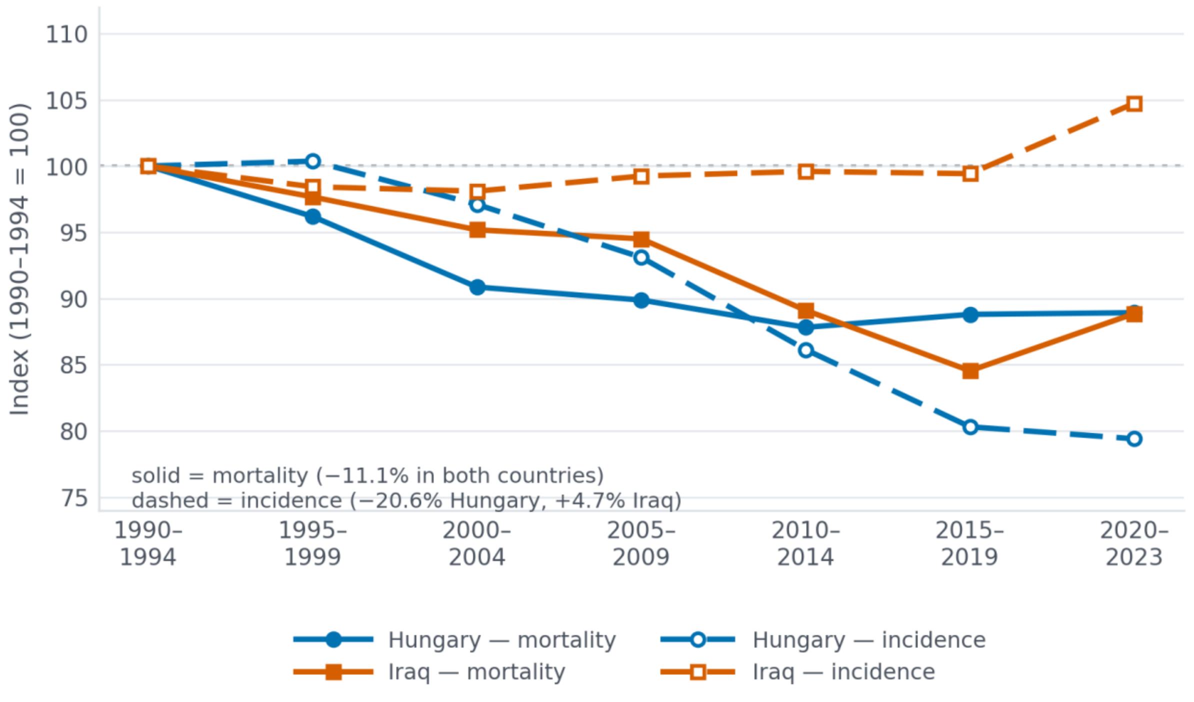

Supplementary Figure S2. Solid lines with filled markers denote mortality, and dashed lines with open markers denote incidence. The two-mortality series end at the same index value, a proportional decline of 11.1% in each country, while the incidence series diverge by approximately 25 index points. Data: GBD 2023, Institute for Health Metrics and Evaluation.

S15. Original crude risk-attributable burden

Table S15. Original crude risk-attributable cardiovascular death and DALY rate changes, 1990–2023

GBD risk factor	Hungary, death rate	Iraq, death rate	Hungary, DALY rate	Iraq, DALY rate
**High body-mass index**	+20.8 (−6.5 to +61.9)	+64.0 (+18.3 to +138.6)	−0.2 (−22.8 to +35.0)	+70.5 (+23.9 to +151.9)
**High fasting plasma glucose**	+3.8 (−19.3 to +34.9)	+35.5 (−0.3 to +91.7)	−8.8 (−29.3 to +20.8)	+57.1 (+12.3 to +121.5)
**Other environmental risks**	+11.3 (+4.3 to +18.4)	+34.9 (+6.6 to +74.6)	-12.8 (−17.8 to −7.6)	+43.4 (+14.3 to +85.0)
**Dietary risks**	-11.1 (−23.9 to −0.2)	+15.8 (−48.3 to +79.3)	-28.6 (−39.5 to −20.3)	+24.8 (−41.3 to +95.6)
**High LDL cholesterol**	-35.6 (−48.2 to −21.4)	+2.3 (−23.7 to +40.0)	-45.3 (−55.7 to −35.4)	+11.2 (−16.5 to +50.4)
**Smoking**	-52.5 (−62.7 to −39.0)	+1.7 (−30.1 to +58.0)	-57.2 (−64.7 to −47.0)	+7.2 (−25.3 to +65.5)
**High systolic blood pressure**	-12.3 (−21.0 to −5.4)	−1.7 (−25.9 to +41.7)	-25.9 (−31.4 to −21.0)	+7.8 (−21.4 to +62.9)
**Non-optimal temperature**	-19.9 (−26.2 to −13.0)	−3.6 (−23.2 to +23.7)	-33.0 (−37.8 to −27.8)	−0.4 (−23.0 to +34.4)
**Kidney dysfunction**	+0.7 (−10.6 to +12.1)	−5.0 (−22.1 to +21.5)	-18.8 (−25.9 to −11.7)	+2.7 (−17.0 to +31.9)
**Low physical activity**	−3.6 (−42.4 to +77.5)	−6.2 (−35.1 to +35.3)	−19.2 (−43.5 to +23.4)	+1.5 (−28.7 to +46.6)
**Diet high in sodium**	−17.9 (−50.5 to +13.1)	−7.3 (−90.0 to +776.3)	-31.9 (−57.1 to −7.6)	+1.0 (−91.4 to +1038.1)
**Air pollution**	-61.7 (−74.1 to −46.1)	−9.2 (−26.7 to +17.6)	-68.0 (−78.1 to −55.2)	−0.9 (−20.8 to +30.5)
**High alcohol use**	−17.4 (−45.5 to +27.4)	−24.4 (−71.0 to +67.9)	−10.3 (−40.6 to +38.6)	−12.3 (−67.9 to +218.2)

Note: Values are the total relative changes in crude, all-age risk-attributable rates from 1990 to 2023. GBD risk-attributable pathways overlap; therefore, risk-specific burdens must not be summed or interpreted as independent causal contributions.

Values are the total relative change between 1990 and 2023, expressed as a percentage, for crude all-age attributable rates. Values in bold indicate uncertainty intervals excluding zero. The rows are ordered by the Iraqi death rate estimate.

Supplementary Table S15 and Supplementary Figure S3 summarize the GBD 2023 estimates of the relative change in cardiovascular death and DALY rates attributable to each major modifiable risk factor between 1990 and 2023. Values are the total proportional change across the 34-year period expressed as a percentage, not an annual rate of change; a value of −52.5% denotes that the attributable rate in 2023 was 52.5% below its 1990 level.

In Hungary, the attributable death rate fell for smoking (−52.5%, 95% UI −62.7 to −39.0), air pollution (−61.7%, −74.1 to −46.1), high LDL cholesterol (−35.6%, −48.2 to −21.4), non-optimal temperature (−19.9%, −26.2 to −13.0), high systolic blood pressure (−12.3%, −21.0 to −5.4), and dietary risks (−11.1%, −23.9 to −0.2), in every case with an uncertainty interval excluding zero. The corresponding declines in attributable DALY rates were larger, reaching −68.0% for air pollution and −57.2% for smoking. Only other environmental risks increased and modestly (+11.3%, +4.3 to +18.4).

In Iraq, the pattern was close to the mirror image. The attributable death rate rose for high body mass index (+64.0%, +18.3 to +138.6) and other environmental risks (+34.9%, +6.6 to +74.6), both with intervals excluding zero, and for high fasting plasma glucose (+35.5%, −0.3 to +91.7), whose interval only marginally includes zero and whose DALY-rate increase is unambiguous (+57.1%, +12.3 to +121.5). No Iraqi risk factor showed a decline with an interval excluding zero. The contrast with Hungary is sharpest for the risks Hungary has most successfully reduced: smoking, which fell by 52.5% in Hungary, was essentially unchanged in Iraq (+1.7%, −30.1 to +58.0), and air pollution, which fell by 61.7% in Hungary, fell by only 9.2% in Iraq (−26.7 to +17.6).

The same divergence is visible in the attributable fractions themselves. The share of Hungarian cardiovascular deaths attributable to smoking fell by 45.5% (−56.6 to −29.3) over the period, whereas the share of Iraqi cardiovascular deaths attributable to high body mass index rose by 75.3% (+34.0 to +137.6).

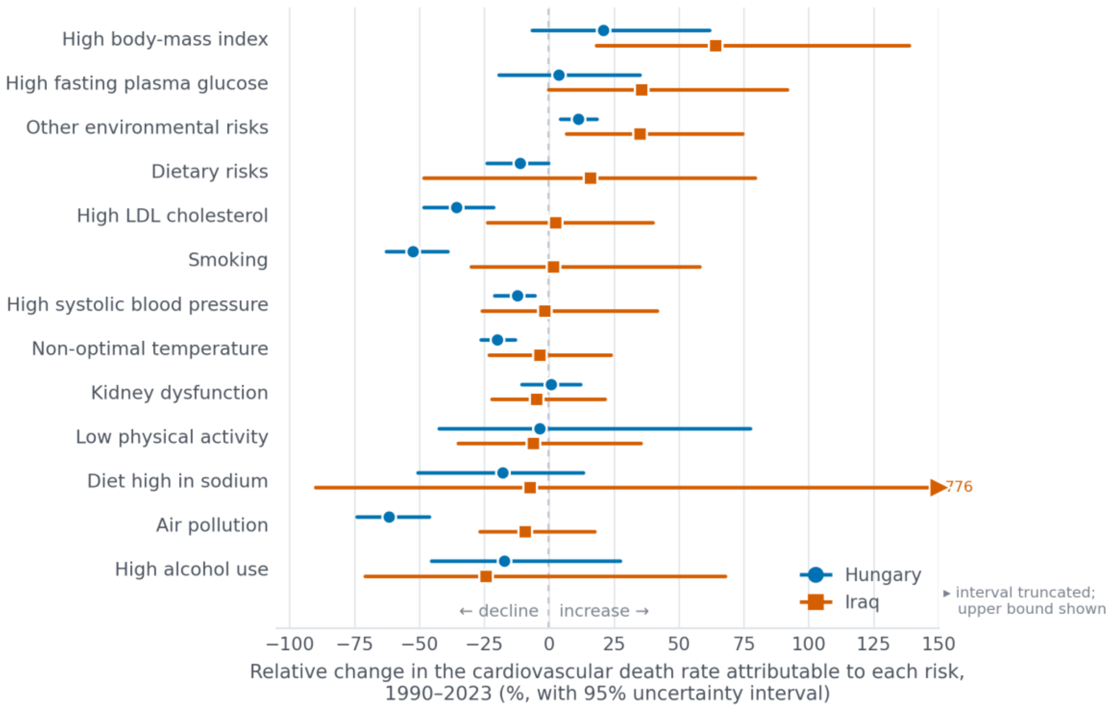

Supplementary Figure S3. Relative changes in cardiovascular death rates attributable to major modifiable risk factors in Hungary and Iraq between 1990 and 2023, with 95% uncertainty intervals. Extremely wide uncertainty intervals extending beyond the displayed axis were truncated for visual clarity; complete values are reported in Supplementary Table S15.

S16. Original crude population-scale risk burden

Table S16. Original crude population-scale risk burden: attributable deaths versus attributable rates, 1990–2023

GBD risk factor	Hungary: number	Hungary: rate	Iraq: number	Iraq: rate	Iraq gap (number − rate)
**High body-mass index**	+11.9	+20.8	+290.6	+64.0	+226.6 pp
**High fasting plasma glucose**	−3.9	+3.8	+222.8	+35.5	+187.3 pp
**Other environmental risks**	+3.1	+11.3	+221.3	+34.9	+186.4 pp
**Dietary risks**	−17.7	−11.1	+175.8	+15.8	+160.0 pp
**High LDL cholesterol**	−40.4	−35.6	+143.7	+2.3	+141.4 pp
**Smoking**	−56.0	−52.5	+142.2	+1.7	+140.5 pp
**High systolic blood pressure**	−18.8	−12.3	+134.0	−1.7	+135.8 pp
**Non-optimal temperature**	−25.8	−19.9	+129.6	−3.6	+133.2 pp
**Kidney dysfunction**	−6.8	+0.7	+126.3	−5.0	+131.3 pp
**Low physical activity**	−10.7	−3.6	+123.5	−6.2	+129.6 pp

**Diet high in sodium**	−23.9	−17.9	+120.8	−7.3	+128.1 pp
**Air pollution**	−64.5	−61.7	+116.1	−9.2	+125.4 pp
**High alcohol use**	−23.5	−17.4	+80.1	−24.4	+104.5 pp

Note: A comparison of attributable death counts with attributable rates illustrates the effect of population change. The difference is descriptive and should not be interpreted as a formal demographic decomposition of the causal effects.

Because the extract reports changes in both the absolute number of attributable deaths and the attributable rate, the two can be compared directly. Their ratio isolates the contribution of population change: for every one of the fourteen risk factors examined, the implied population factor is identical to four decimal places within each country, 0.9259 for Hungary (a population decline of 7.4%) and 2.3816 for Iraq (a population increase of 138.2%).

The consequences are set out in Supplementary Table S16 and Supplementary Figure S4. In Hungary, changes in the number of attributable deaths closely tracked changes in the rate, differing by 6–8 percentage points across all risks because the population barely changed. In Iraq, the two diverge by 105–227 percentage points. Attributable deaths from high body mass index rose by 290.6% in number but by 64.0% in rate; from high fasting plasma glucose, by 222.8% in number but 35.5% in rate; and from high systolic blood pressure, by 134.0% in number while the rate fell slightly (−1.7%).

Iraq's absolute cardiovascular risk burden grew steeply for every risk factor examined, including those whose per-capita rate was flat or falling, and the great majority of this growth is demographic rather than epidemiological. It also has a direct operational implication: a health system must treat the number of cases, not the rate; thus, Iraq faces a rapidly expanding absolute burden even where per-capita risk is stable.

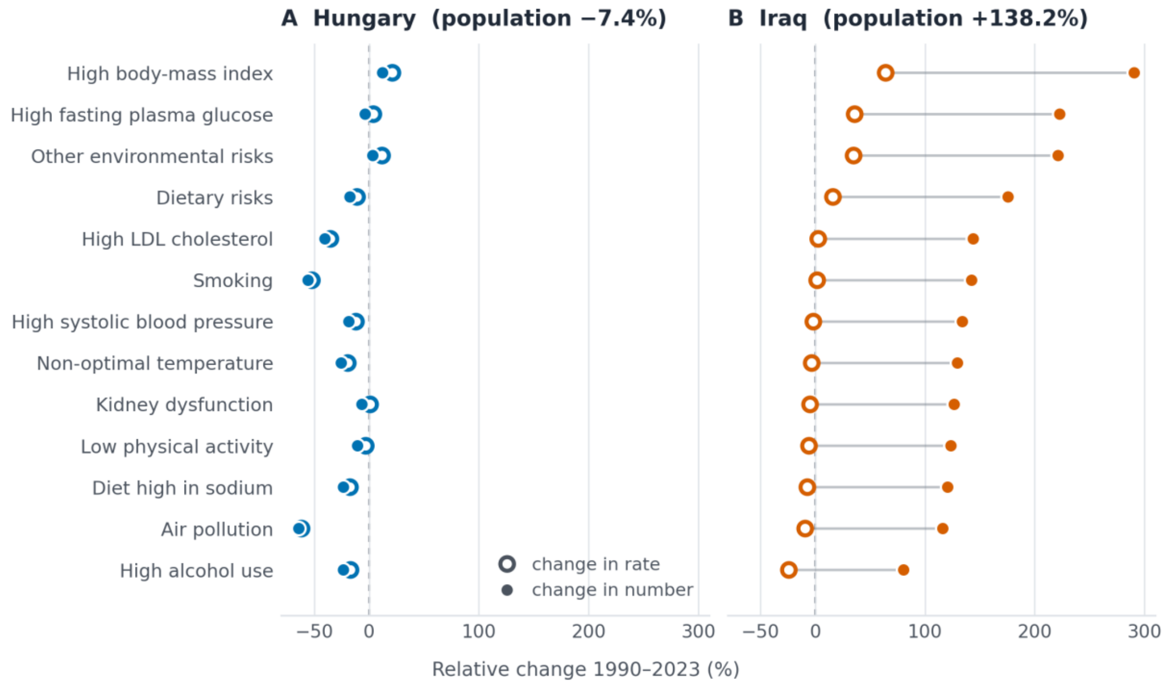

Supplementary Figure S4. Relative change in attributable cardiovascular deaths, 1990–2023, shown as change in rate (open circles) and change in number (filled circles) for each risk factor. (A) Hungary, where the population fell by 7.4%, and the two measures nearly coincide. (B) Iraq, where the population rose by 138.2%, and the two diverge by 105–227 percentage points.

S17. Original crude mortality-to-incidence ratios

Table S17. Original crude mortality-to-incidence ratios by cause, 1990–2023

Cause	Hungary MIR	Iraq MIR	Iraq ÷ Hungary
**All cardiovascular disease**	0.476	0.445	0.94
**Ischaemic heart disease**	0.535	0.503	0.94
**Stroke**	0.698	0.697	1.00
**Atrial fibrillation and flutter**	0.063	0.059	0.94
**Pulmonary arterial hypertension**	0.448	0.400	0.89
**Non-rheumatic valvular heart disease**	0.092	0.175	1.89
**Endocarditis**	0.060	0.027	0.46
**Cardiomyopathy and myocarditis**	2.165	0.426	0.20
**Rheumatic heart disease**	0.654	0.042	0.06
**Lower-extremity peripheral arterial disease**	0.033	0.0004	0.01

Note: These original crude MIR estimates were retained for sensitivity analysis only. They should not be interpreted as case fatality probabilities; causes with substantially different incidence and mortality time scales require particular caution.

Supplementary Table S17 and Supplementary Figure S5 report the mortality-to-incidence ratio for each cause with both measures available (n = 9 component causes plus the aggregate). For the three causes that carry the burden, the two countries show closely similar ratios: all cardiovascular diseases, 0.476 in Hungary versus 0.445 in Iraq; ischemic heart disease, 0.535 versus 0.503; and stroke, 0.698 versus 0.697. The broadly similar mortality-to-incidence ratios for the dominant cardiovascular causes suggest that differences in incidence may contribute more strongly to the observed between-country contrast than do differences in mortality. However, these ratios are ecological proxies and should not be interpreted as direct measures of mortality.

Three causes behave anomalously and are reported but excluded from interpretation, for reasons that also explain why the cross-cause correlation examined in Section S18 is not an appropriate analysis. Iraq's rheumatic heart disease (0.042) and lower-extremity peripheral arterial disease (0.0004) ratios reflect a young incident population whose deaths are deferred by decades, compounded by sparse underlying data. Hungary's cardiomyopathy ratio exceeds 1.0 (2.165) with more deaths per year than incident cases because those deaths arise from a long-accumulated prevalent pool. Cross-sectional MIR is therefore a proxy rather than a measure of case fatality and is informative only where incidence and mortality operate on comparable timescales.

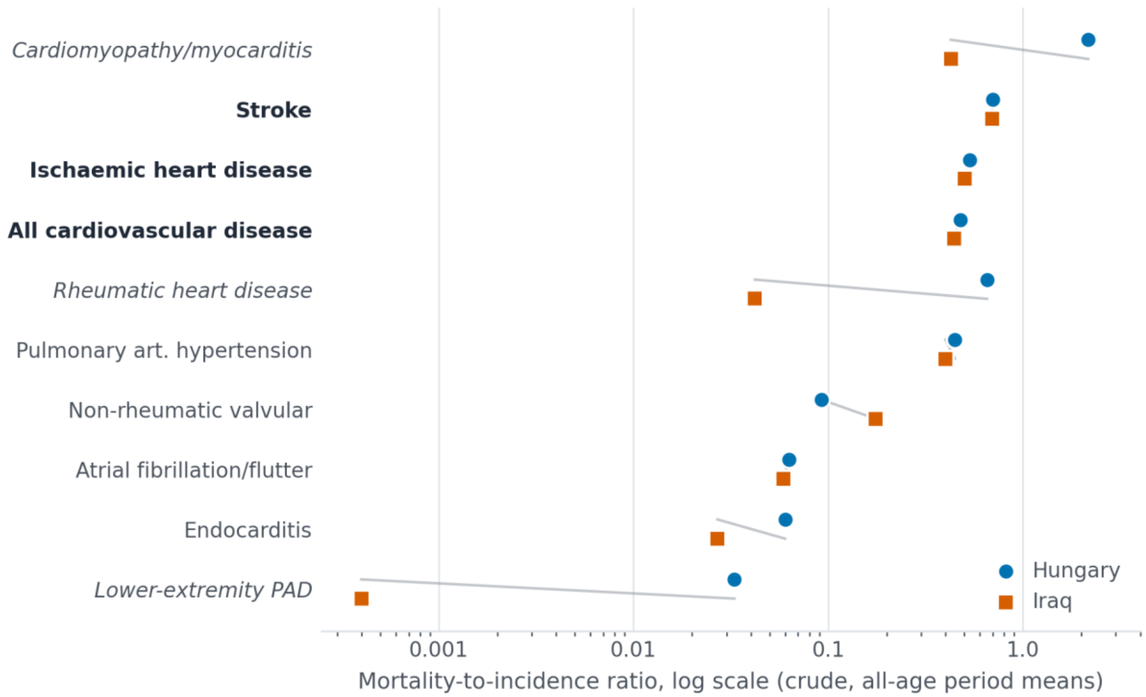

Supplementary Figure S5. Mortality-to-incidence ratio by cause in Hungary and Iraq on a logarithmic scale. The causes shown in bold type, cardiovascular disease, ischemic heart disease, and stroke, have closely similar ratios in both countries. The causes in italics have incidence and mortality rates that operate on non-comparable timescales and are excluded from the interpretation. Ratios were computed from crude, all-age means.

S18. Original crude cross-cause correlation between incidence and mortality

Cause-specific incidence and mortality were positively associated in both countries, but the association was weak and reported here only for completeness. Because the rates spanned more than three orders of magnitude (0.02 to 351.68 per 100,000), Pearson 'scorrelation required log transformation. On the log scale, the association was r = 0.782 in Hungary ($p = 0.013$, $R^2 = 0.611$) and r = 0.559 in Iraq ($p = 0.118$, $R^2 = 0.312$, not significant), with Spearman ρ = 0.750 and 0.667, respectively.

The result is driven by very few points: omitting ischemic heart disease reduces the Iraqi coefficient from 0.559 to 0.397, and omitting pulmonary arterial hypertension reduces the Hungarian coefficient from 0.782 to 0.621. With n = 9 causes, this is a nine-point scatter rather than a population-level finding, and a positive association is close to tautological because both measures scale with the commonality of a disease. The mortality-to-incidence ratio reported in Section S17 uses the same data to address a question with an epidemiological interpretation and supersedes this analysis.

S19. Data, code, and reproducibility

The complete annual machine-readable disease dataset, selected CVD risk factor dataset, model output tables, annual fitted curves, turning points, data validation results, source provenance, and statistical syntax are provided in the accompanying Excel workbook and code file. Source exports were supplied by the GBD 2023 Results Tool and retained in the reviewer package.

GBD source: Global Burden of Disease Collaborative Network. Global Burden of Disease Study 2023 (GBD 2023) Results. Seattle, United States: Institute for Health Metrics and Evaluation (IHME). GBD Results Tool: https://vizhub.healthdata.org/gbd-results/.
